# Knowledge, attitude, and practices of stakeholders involved in healthcare financing programs on economic evaluations in Cameroon

**DOI:** 10.1371/journal.pgph.0003101

**Published:** 2024-04-25

**Authors:** Eric Tchouaket, Katya Kruglova, Isidore Sieleunou, Marcellin Tsafack, Joseph Maabo Tankwa, Gislaine Takoguen, Nikolas Argiropoulos, Stephanie Robins, Drissa Sia

**Affiliations:** 1 Department of Nursing, Université du Québec en Outaouais, Saint-Jérôme, Québec, Canada; 2 Research for Development International, Yaoundé, Cameroon; 3 Medical Department, Médecins Sans Frontières, Yaoundé, Cameroon; 4 Department of Mathematics and Statistics, Université de Montréal, Montréal, Québec, Canada; Sheffield Hallam University, UNITED KINGDOM

## Abstract

There are many healthcare financing programs (HFPs) in Cameroon; however, there is a lack of information on these programs’ economic effectiveness and efficiency. Involvement of local stakeholders in the economic evaluations (EEs) of HFPs is critical for ensuring contextual factors are considered prior to program implementation. We conducted a cross-sectional study to assess the need for EEs of Cameroonian HFPs. Regular staff in supervisory roles aged 18 years and above were recruited in four Cameroonian cities. Data were collected via face-to-face surveys between June 15 and August 1, 2022. Descriptive analyses summarized participants’ knowledge, attitudes, and practices in relation to performing EEs of HFPs. Principal component analyses identified organizational, individual, and contextual factors that could influence participants’ involvement. The total sample included 106 participants. On average, 65% of participants reported being aware of the listed HFPs; however, of these, only 28% said that they had been involved in the HFPs. Of the 106 participants, 57.5% knew about EEs; yet, almost 90% reported that the HFP in question had never been subject to an EE, and 84% had never been involved in an EE. Most participants indicated that they had intended or would like to receive EE training. Using principal component analyses, the organizational factors were classified into two components (‘policy and governance’ and ‘planning and implementation’), the individual factors were classified into two components (‘training’ and ‘motivation’), and the contextual factors were classified into three components (‘funding,’ ‘political economy,’ and ‘public expectations’). The findings of this study highlight the need to invest in EE training to improve participation rates of Cameroonian stakeholders in the EEs of HFPs. Improved knowledge, diversified skills, and increased participation of stakeholders from all levels of the Cameroonian healthcare system are critical to the effective and efficient development, implementation, and EE of the country’s HFPs.

## Introduction

Cameroon is a lower-middle-income country that ranks 151 out of 191 countries on the 2021 Human Development Index, which measures countries’ achievement in the dimensions of health, education, and standard of living [1]. In 2018, almost 45% of Cameroonians were poor in terms of health, education, and standard of living, as captured by the Multidimensional Poverty Index [2]. Life expectancy at birth in Cameroon is 60.3 years [3], compared to the world average of 73.2 years [4]. HIV/AIDS, malaria, diarrheal diseases, and lower respiratory tract infections remain the main causes of mortality in the country, along with an increase in non-communicable diseases, such as diabetes and cardiovascular disease [5,6].

In 2020, Cameroon’s health expenditure as a percentage of Gross Domestic Product was 3.8, compared to 4.9 across Sub-Saharan Africa, 12.9 in Canada, and 18.8 in the United States of America (USA) [7]. In the same year, Cameroonian patients paid for almost 70% of total healthcare costs out-of-pocket, a system that renders care inaccessible for many citizens [6]. By contrast, in 2020 out-of-pocket expenditure as a percentage of total health expenditure was 30.4% across Sub-Saharan Africa, 12.4% in Canada, and 9.9% in the USA [7]. The distribution of human resources within the Cameroonian healthcare system is characterized by geographic inequalities, with a concentration of care delivery in urban centers and critical shortages of staff in rural areas [6].

In such a complex context, health policy makers in Cameroon face multiple challenges, such as: 1) how to optimize the use of the existing healthcare resources to ensure the accessibility, safety, and quality of care, 2) how to prioritize healthcare interventions, and 3) how to integrate available economic data into the budget for health financing programs (HFPs). HFPs are sets of processes for collecting and distributing funds allocated towards the provision of healthcare services [8,9]. Stated differently, HFPs are ‘types of financial arrangements through which health services are paid for and obtained by people,’ such as social health insurance, government insurance, and out-of-pocket household payments [10].

Few studies have assessed the economic effectiveness and efficiency of different HFPs [11,12]. Some reasons for this may include unavailable funding, insufficient organization and planning, or a lack of training in conducting economic evaluations (EEs). Collaboration between diverse stakeholders, including researchers, healthcare professionals (HCPs), managers, and policy makers can facilitate the use of economic data in real-world settings [13]. Since health systems are complex multi-tier networks, local input during the conception phase of an EE is critical for ensuring that relevant contextual factors are considered prior to implementation [14]. On the other hand, failure to integrate knowledge about political, economic, and sociocultural contexts of an HFP into an EE can hinder the program’s effectiveness [15]. Determining best ways of integrating data from EEs into current organizational processes necessitates insight into the gap between the current and desired knowledge, attitudes, and practices of Cameroonian stakeholders toward conducting and using EEs of HFPs.

Accordingly, the main objective of our study was to assess the need for EEs of HFPs, as expressed by diverse stakeholders employed at various levels of the Cameroonian healthcare system. Specifically, we aimed to examine the knowledge, attitude, and practices of stakeholders with respect to conducting EEs of the existing HFPs in Cameroon. In addition, we aimed to identify organizational, individual, and contextual factors that could influence the involvement of stakeholders in the EEs of Cameroonian HFPs.

## Literature review

### Economic evaluation

An EE involves comparing the effects of a program or policy with the costs incurred during its implementation. An EE provides public health officials, managers, and HCPs with information on the economic consequences of a policy [16]. It also assesses the impact of a program by answering the question: In the absence of a program, would the situation be different from another program or the status quo? Stated differently, during an EE the effects of a policy are compared before and after its implementation, or more accurately, in the presence and absence of the policy. An EE gauges the efficiency of a program in relation to the costs generated during its implementation, thus estimating the program’s effectiveness, or its return on investment [17].

### Types of economic evaluations

There are five major types of EEs commonly used to determine the effectiveness of a program or policy: cost-minimization analysis (CMA), cost-effectiveness analysis (CEA), cost-utility analysis (CUA), cost-benefit analysis (CBA), and cost-consequences analysis (CCA) [18–22]. CMA compares the costs of implementing a program, while assuming identical outcomes, to identify the least expensive program. CEA determines the most cost-effective program (expressed in cost per years of life gained) among programs with varying levels of effectiveness. In a CUA, the benefits of a program are adjusted to signify the value of the years of life gained and expressed in quality-adjusted life-years (QALYs). Here, the most efficient program is identified by estimating the differential cost-utility ratio, which represents the additional costs necessary for an increase in QALYs. In a CBA, the benefits and costs of a program are expressed in monetary units to estimate a net gain or loss. The program’s return on investment is measured by comparing the program to the status quo. CCA involves compiling a table with estimated costs and potential outcomes to provide decision makers with information on the relative importance of specific outcomes.

### Health financing programs in Cameroon

The inclusion of HFPs in our study was based on a scoping review by Sieleunou et al. [23]. This review identified 30 existing HFPs in Cameroon that can be grouped into the following nine categories: 1) free/subsidy policy focusing on disease control for the entire population, 2) free/subsidy policy focusing on controlling a disease targeting part of population, 3) free care for services, 4) free care for indigents, 5) budget financing, 6) budget support targeting a segment of the population, 7) prepayment mechanism, 8) results based financing, and 9) payment at the point of service. Table 1 provides a complete list of the HFPs.

**Table 1:** Healthcare financing programs in Cameroon.

**Free/subsidy policy focusing on disease control for the entire population**	1. Subsidized treatment for diabetes 2. Free care for epilepsy 3. Free care for preventive treatment of onchocerciasis 4. Free care for HIV/AIDS 5. Free care for tuberculosis 6. Free care for leprosy 7. Free care for Buruli ulcer 8. Subsidized treatment for cancer
**Free/subsidy policy focusing on controlling a disease targeting part of the population**	9. Free malaria treatment for children under 5 years old 10. Subsidized malaria treatment for children over 5 years old and adults 11. Free intermittent preventive treatment (IPT) for pregnant women 12. Free long lasting insecticidal (LLI) bed nets 13. Free care for malnutrition 14. Free treatment for intestinal helminthiasis 15. Free care for schistosomiasis 16. Free care for diabetes (0–18 years)
**Free care for services**	17. Free care for family planning
**Free care for indigents**	18. Free care for abandoned children 19. Free care for indigents
**Budget financing**	20. Subvention for care in confessional facilities 21. Budget support for public health facilities
**Budget support targeting a segment of the population**	22. Medical evacuation funds (abroad) 23. Subsidized care for civil servants and health personnel
**Prepayment mechanism**	24. National health insurance 25. Social security 26. Private health insurance 27. Mutual health organization
**Results based financing**	28. Voucher 29. Performance based financing
**Payment at the point of service**	30. Out of pocket payment

*Note*: Adopted from Sieleunou et al. [23].

### Conceptual framework

**Fig 1:**
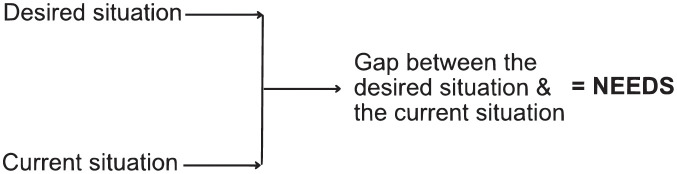
Conceptual framework. Adapted from Lapointe (1992) [24].

This study’s conceptual framework is based on Lapointe’s [24] concept of needs, where the gap signifies the difference between the current situation and the desired situation (see Fig 1). In the healthcare context, these needs represent the gap between HCPs’ current skills and the skills that they would like to possess [25]. Thus, this framework focuses on the discrepancies between HCPs’ current and desired knowledge, attitude, and practices in relation to a phenomenon of interest.

## Methods

### Ethics statement

This study was approved by the National Ethics Committee for Human Health Research in Cameroon (*Comité National d’Éthique de la Recherche pour la Santé Humaine*) [2021/07/1736/L/CNERSH/SP]. All participants provided informed consent prior to participating in the study.

### Inclusivity in global research

Additional information regarding the ethical, cultural, and scientific considerations specific to inclusivity in global research is included in S1 Checklist.

### Study design, setting, and participants

We conducted a cross-sectional study in four cities in Cameroon: Bafoussam, Bamenda, Douala, and Yaoundé. The aim was to recruit a diverse sample of stakeholders involved within the field of HFPs, for example members of the Ministry of Health, country-level donors, and representatives from health insurance companies, research institutes, pharmaceutical companies, and non-governmental organizations. Individuals were eligible to participate if they were at least 18 years old and employed as regular staff in supervisory roles (e.g. director, CEO) within HFPs.

### Sample size and recruitment

Of the 111 people who had been deemed eligible and targeted, 106 consented and participated in the survey (95.5% response rate). We had not performed power calculations prior to the study because our goal was to recruit all regular staff in supervisory roles who were involved within Cameroonian HFPs. Purposive and snowball sampling methods were used [26], where regional health officials were consulted to determine initial key informants who then suggested other potential participants. These non-probability sampling methods were employed because in Cameroon reaching and recruiting staff in supervisory roles for research purposes presents a major challenge. Eligible individuals were contacted by email or phone to discuss the study and arrange a date/time for a face-to-face survey. Recruitment took place between June 15 and August 1, 2022.

### Data collection

Data were collected between June 15 and August 1, 2022 via face-to-face surveys. The surveys were conducted in either French or English. Both versions are included in S1 Questionnaire. Participant responses were inputted into an electronic database set up on Google Forms.

The questions were developed by our team based on the knowledge, attitude, and practices (KAP) survey model and subsequently validated during a meeting with six co-authors (ET, IS, MT, JMT, GT, and DS). KAP surveys are widely used in the field of public health to collect information about ‘what is known, believed, and done’ in relation to a phenomenon of interest [27]. Interview questions were grouped into five sections from A—F (see S1 Questionnaire). In section A, participants were asked about their socio-demographic characteristics, such as their place of residence and years of professional experience. Section B inquired about participants’ knowledge of and level of involvement in HFPs. Section C collected information on participants’ awareness of different types of EEs and their application. Section D asked participants about their current practices in relation to EEs. In section E, participants were asked if they were interested in getting involved in an EE of an HFP, whether they wished to receive EE training, and if so, which form of training they would prefer (e.g. face-to-face workshop, online conference). Section F collected participants’ opinion on what could influence stakeholder participation in an EE of an HFP. Participants were presented with the statement: ‘Factor __ could influence people’s participation in an EE of HFPs,’ which was followed by a list of 27 factors (e.g. political dynamics, level of training). Participants were asked to state their agreement with the statement for each of the listed factors using a Likert scale, from 1–strongly disagree to 5–strongly agree. All factors were classified into organizational (10 factors), individual (6 factors), or contextual (11 factors).

### Statistical analyses

First, we ran descriptive analyses (frequencies, means, medians, standard deviations) to examine participants’ knowledge, attitude, and practices in relation to conducting EEs of the existing HFPs in Cameroon. Second, we performed a principal component analysis (PCA) for each of the three categories of factors presented in section F (organizational, individual, contextual) to identify sets of factors that could influence stakeholder participation in an EE of an HFP. PCAs were used to transform the three sets of inter-correlated variables into three sets of new variables called principal components [28]. Kaiser-Meyer-Olkin (KMO) tests, Bartlett’s tests, and determinant values were used to verify the appropriateness of the PCA. The 0.3 cut-off was applied to all loadings [29]. Three contextual factors were removed due to high cross-loadings. After the determination of the number of components, a varimax rotation was used for the contextual factors, and a direct oblimin rotation was used for the institutional and individual factors due to multiple variables having high loadings on more than one factor. The statistical software SPSS version 28 was employed for all analyses.

## Results

### Socio-demographic characteristics

The final sample included 106 participants, whose socio-demographic characteristics are presented in Table 2. There were slightly more males (54.7%, 58/106) than females (45.3%, 48/106). Most participants were employed by the Ministry of Health including regional and district offices (35.8%, 38/106), hospitals (31.1%, 33/106), or health facilities other than hospitals such as health centres and medical clinics (14.2%, 15/106). The four cities were roughly equally represented, with the most participants (32.1%, 34/106) residing in Douala, Cameroon’s largest city. The average participant age was 41.0 years (SD = 8.0, range = 26–58), the average professional experience was 12.1 years (SD = 7.3, range = 1–31), and the average duration of involvement with the current organization or program was 5.0 years (SD = 4.0, range = 1–22).

**Table 2:** Participants’ socio-demographic characteristics (N = 106).

	Frequency (%)	Mean (SD)(Range)
**Sex**		
Female	48 (45.3)	
Male	58 (54.7)	
**Organization**		
Ministry of Health (including regional and district offices)	38 (35.8)	
Hospitals	33 (31.1)	
Health facilities other than hospitals (e.g., health centres, medical clinics)	15 (14.2)	
Non-governmental organizations	6 (5.7)	
Academic institutions	4 (3.8)	
International institutions	1 (0.9)	
Other (e.g., pharmacies, insurance companies)	9 (8.5)	
**City**		
Douala	34 (32.1)	
Yaoundé	33 (31.1)	
Bafoussam	23 (21.7)	
Bamenda	16 (15.1)	
**Age** (years)		40.99 (7.99)(26–58)
**Professional experience** (years)		12.06 (7.26)(1–31)
**Experience with the organization/program** (years)		4.99 (3.96)(1–22)

### Knowledge of and level of involvement in HFPs

Table 3 summarizes participants’ knowledge of, and level of involvement in, each of the nine categories of the HFPs (see S1 Table for complete data). One participant was deemed a careless responder; their data were removed from the analyses, resulting in the sample of 105. The average level of knowledge across all nine categories of HFPs was 65.4% (68.7/105). Among the participants who reported being aware of the HFPs, the average level of involvement was 28.1%. When considering only those who reported some level of involvement, a greater proportion (50.3%) of stakeholders had been involved in the implementation of a program on the ground, relative to the other levels of involvement, such as evaluation (22.0%) or theoretical design (10.8%).

**Table 3:** Participants’ knowledge of and level of involvement in Cameroon’s healthcare financing programs (N = 105).

Healthcare financing programs (n = number of programs in the category)	Do you know this program? n (% selected)	Some level of involvement—n (% selected out of those who know the program)	Level of involvement—n (% selected out of those who indicated some level of involvement)					
			Theoretical design	Design in terms of consultation/ discussion with funders or funding policy	Implementation in terms of program coordination or funding policy	Implementation on the ground	Evaluation	Program improvement
Free/subsidy policy focusing on disease control for the entire population (**n = 8**)	63.8 (60.7)	20.3 (40.5)	1.1 (4.0)	1.6 (6.0)	2.4 (10.3)	13.3 (54.4)	5.9 (23.4)	2.6 (8.8)
Free/subsidy policy focusing on controlling a disease targeting part of the population (**n = 8**)	73.4 (69.9)	26.0 (34.7)	1.8 (6.0)	3.3 (11.6)	4.6 (16.7)	19.5 (70.5)	8.4 (30.3)	3.5 (12.2)
Free care for services (**n = 1**)	75.0 (71.4)	29.0 (38.7)	1.0 (3.4)	1.0 (3.4)	1.0 (3.4)	23.0 (79.3)	8.0 (27.6)	1.0 (3.4)
Free care for indigents (**n = 2**)	58.0 (55.2)	17.5 (30.4)	2.0 (11.5)	1.0 (5.8)	2.0 (11.5)	11.0 (62.3)	3.0 (17.3)	1.0 (5.8)
Budget financing (**n = 2**)	54.5 (51.9)	9.5 (19.3)	1.5 (15.3)	2.5 (26.1)	2.5 (27.8)	4.5 (49.4)	2.5 (26.1)	2 (21.6)
Budget support targeting a segment of the population (**n = 2**)	58.5 (55.7)	10.0 (17.1)	2.0 (19.2)	1.5 (14.6)	1.5 (14.6)	2.5 (24.7)	1.5 (14.6)	1.5 (14.6)
Prepayment mechanism (**n = 4**)	73.0 (69.5)	14.5 (19.6)	2.0 (16.4)	1.5 (9.8)	0.8 (5.7)	5.0 (29.6)	1.8 (12.3)	1.5 (9.8)
Results based financing (**n = 2**)	76.0 (72.4)	25.0 (31.0)	3.0 (10.6)	4.0 (15.8)	4.5 (14.7)	15.0 (45.5)	8.5 (30.5)	4.5 (14.7)
Payment at the point of service (**n = 1**)	86.0 (81.9)	19.0 (22.1)	2.0 (10.5)	3.0 (15.8)	2.0 (10.5)	7.0 (36.8)	3.0 (15.8)	3.0 (15.8)
**Overall Mean**	**68.7 (65.4)**	**19.0 (28.1)**	**1.8 (10.8)**	**2.2 (12.1)**	**2.4 (12.8)**	**11.2 (50.3)**	**4.7 (22.0)**	**2.3 (11.9)**

### Involvement in economic evaluation

Of the 106 participants, 80.2% (85/106) reported having never participated in an EE, whereas 65.1% (69/106) said that someone had been assigned to perform EEs in their organization. Of these 69 participants, 40.6% (28/69) reported that the evaluation had been performed, 36.2% (25/69) said it had not, and the rest did not know.

Table 4 summarizes participants’ level of involvement in an EE of each of the nine categories of the HFPs (see S2 Table for complete data). On average, only 15.8% of participants (16.8/106) reported some level of involvement in an EE of the listed HFPs.

**Table 4:** Participants’ level of involvement in a health economic evaluation of Cameroon’s healthcare financing programs (N = 106).

Healthcare financing programs (n = number of programs in the category)	Some level of involvement—n (% selected)	Level of involvement in a health economic evaluation—n (% selected out of those who indicated some level of involvement)					
		Initial and theoretical design	Design in terms of consultation/ discussion with donors	Implementation in terms of coordination	Implementation on the ground	Data analysis	Improvement
Free/subsidy policy focusing on disease control for the entire population (**n = 8**)	19.0 (17.9)	0.3 (1.6)	0.3 (1.6)	0.0 (0.0)	0.3 (1.6)	0.3 (1.6)	0.0 (0.0)
Free/subsidy policy focusing on controlling a disease targeting part of the population (**n = 8**)	15.5 (14.7)	0.3 (2.1)	0.3 (2.1)	0.3 (2.1)	0.3 (2.1)	0.3 (2.1)	0.3 (2.1)
Free care on services (**n = 1**)	0.0 (0.0)	-	-	-	-	-	-
Free care for indigents (**n = 2**)	18.5 (17.5)	1.0 (5.5)	1.0 (5.5)	0.0 (0.0)	0.0 (0.0)	0.0 (0.0)	0.0 (0.0)
Budget financing (**n = 2**)	20.0 (18.9)	0.0 (0.0)	0.0 (0.0)	0.0 (0.0)	0.0 (0.0)	0.0 (0.0)	0.0 (0.0)
Budget support targeting a segment of the population (**n = 2**)	19.5 (18.4)	0.0 (0.0)	0.0 (0.0)	0.0 (0.0)	0.0 (0.0)	0.0 (0.0)	0.0 (0.0)
Prepayment mechanism (**n = 4**)	20.3 (19.1)	0.0 (0.0)	0.0 (0.0)	0.0 (0.0)	0.0 (0.0)	0.0 (0.0)	0.0 (0.0)
Results based financing (**n = 2**)	17.0 (16.1)	0.5 (2.7)	0.0 (0.0)	0.0 (0.0)	0.0 (0.0)	0.0 (0.0)	0.0 (0.0)
Payment at the point of service (**n = 1**)	21.0 (19.8)	0.0 (0.0)	0.0 (0.0)	0.0 (0.0)	0.0 (0.0)	0.0 (0.0)	0.0 (0.0)
**Overall Mean**	**16.8 (15.8)**	**0.3 (1.5)**	**0.2 (1.1)**	**0.04 (0.3)**	**0.1 (0.5)**	**0.1 (0.5)**	**0.04 (0.3)**

### Knowledge of economic evaluation

Of the 106 participants, 61 (57.5%, 61/106) had heard about EEs. Of these 61 participants, a large majority (93.4%, 57/61) thought that their organization should play a role in the implementation of EEs, and 86.9% (53/61) thought that other organizations should also be involved. Of these 61 participants, most had learned about EEs in university (49.2%, 30/61), at a seminar or training workshop (37.7%, 23/61), from healthcare personnel (23.0%, 14/61), or via radio or television (23.0%, 14/61).

Of these 61 participants, the majority of participants thought that EEs could be used for making decisions (78.7%, 48/61), allocating resources (67.2%, 41/61), determining the effectiveness of an intervention (65.6%, 40/61), generating cost estimates (63.9%, 39/61), and gauging the efficiency of an intervention (54.1%, 33/61). Most participants reported being aware of cost-analysis (73.8%, 45/61), cost-effectiveness analysis (72.1%, 44/61), and cost-benefit analysis (59.0%, 36/61). Cost-utility analysis and cost-consequences analysis were known to less than half of participants, 36.1% (22/61) and 24.6% (15/61), respectively. No one reported having knowledge of cost-minimization analysis, and 8.2% (5/61) said that they did not know any of the analyses.

Table 5 summarizes participants’ reported knowledge of whether any of the nine categories of Cameroonian HFPs had been subject to an EE (see S3 Table for complete data). Most (97.6%) reported that the HFP in question had never been subject to an EE nor was one planned. Relative to the other EE types, a greater proportion of participants (72.8%) said that the HFP had been subject to a cost analysis.

**Table 5:** Participants’ knowledge of whether Cameroon’s healthcare financing programs have been the subject of economic evaluation (N = 106).

Healthcare financing programs (n = number of programs in the category)	Do not know if the program has ever been the subject of economic evaluation, or if it is planned—n (% selected)	Has never been the subject of economic evaluation, and it is not planned—n (% selected out of those who know if the program has ever been the subject of economic evaluation, of it is planned)	Has already been the subject of … n (% selected out of those who indicated that the program has been the subject of economic evaluation)						
			Cost analysis	Effects/ consequences analysis	Cost -minimization analysis	Cost-effectiveness analysis	Cost-utility analysis	Cost-benefit analysis	Cost-consequences analysis
Free/subsidy policy focusing on disease control for the entire population (**n = 8**)	8.3 (7.8)	94.1 (96.3)	2.6 (76.1)	0.3 (8.8)	0.0 (0.0)	0.1 (2.5)	0.0 (0.0)	0.0 (0.0)	0.0 (0.0)
Free/subsidy policy focusing on controlling a disease targeting part of the population (**n = 8**)	11.4 (10.7)	92.3 (97.5)	2.3 (95.8)	0.6 (25.0)	0.0 (0.0)	0.5 (20.8)	0.0 (0.0)	0.0 (0.0)	0.0 (0.0)
Free care on services (**n = 1**)	9.0 (8.5)	94.0 (96.9)	2.0 (66.7)	2.0 (66.7)	0.0 (0.0)	0.0 (0.0)	0.0 (0.0)	0.0 (0.0)	0.0 (0.0)
Free care for indigents (**n = 2**)	7.0 (6.6)	97.0 (98.0)	2.0 (100.0)	0.0 (0.0)	0.0 (0.0)	0.0 (0.0)	0.0 (0.0)	0.0 (0.0)	0.0 (0.0)
Budget financing (**n = 2**)	8.5 (8.0)	95.5 (98.0)	1.0 (33.4)	0.0 (0.0)	0.0 (0.0)	0.0 (0.0)	0.0 (0.0)	0.0 (0.0)	0.0 (0.0)
Budget support targeting a segment of the population (**n = 2**)	7.0 (6.6)	96.5 (97.5)	1.5 (58.4)	0.0 (0.0)	0.0 (0.0)	0.0 (0.0)	0.0 (0.0)	0.0 (0.0)	0.0 (0.0)
Prepayment mechanism (**n = 4**)	8.0 (7.6)	96.5 (98.5)	1.3 (91.7)	0.0 (0.0)	0.0 (0.0)	0.0 (0.0)	0.0 (0.0)	0.0 (0.0)	0.0 (0.0)
Results based financing (**n = 2**)	13.5 (12.8)	91.5 (99.0)	1.0 (100.0)	0.0 (0.0)	0.0 (0.0)	0.0 (0.0)	0.0 (0.0)	0.0 (0.0)	0.0 (0.0)
Payment at the point of service (**n = 1**)	9.0 (8.5)	94.0 (96.9)	1.0 (33.3)	0.0 (0.0)	0.0 (0.0)	0.0 (0.0)	0.0 (0.0)	0.0 (0.0)	0.0 (0.0)
**Overall Mean**	**9.1 (8.6)**	**94.6 (97.6)**	**1.6 (72.8)**	**0.3 (11.2)**	**0.0 (0.0)**	**0.1 (2.6)**	**0.0 (0.0)**	**0.0 (0.0)**	**0.0 (0.0)**

### Attitude towards performing economic evaluation

The majority of participants said that they had intended or would like to be involved in an EE, with the percentages ranging from 92.5% to 95.3% (see Table 6). Most (94.3%, 100/106) said they had intended or would like to receive training in conducting EEs. Of these, most would prefer to receive training in the form of a face-to-face seminar or workshop (85.0%, 85/100) or an in-person conference (59.0%, 59/100).

**Table 6:** Participants’ attitude towards performing an economic evaluation of healthcare financing programs (N = 106).

Intend / would like to participate in the …	Frequency (%)
… theoretical design of an EE of an HFP	101 (95.3)
… design of an EE of an HFP in terms of consultation/discussion with donors	99 (93.4)
… implementation of an EE of an HFP in terms of coordination	99 (93.4)
… implementation of an EE of an HFP on the ground	101 (95.3)
… data analysis of an EE of an HFP	98 (92.5)
… improvement of an HFP based on the results of an EE	100 (94.3)
… training in health EE	100 (94.3)

EE = economic evaluation.

HFP = healthcare financing program.

### Factors influencing involvement in economic evaluation

A PCA was conducted for each of the three categories of factors (organizational, individual, contextual) to identify factors that participants thought could influence stakeholder involvement in the EEs of the HFPs. Results of the PCAs are presented in Table 7.

**Table 7:** Principal component analysis of factors influencing stakeholder involvement in Cameroon’s healthcare financing programs.

**Organizational factors**	**N**	KMO* **(Determinant)**	**Cumulative variance**	**Loadings** **after rotation**
	103	0.873 (0.006)	61.88%	
**Component 1: Policy and governance**				
Political will of the decision-maker				0.879
Governance and priority of decision-makers				0.816
Collaboration between the local and regional/central administration				0.720
Leadership				0.587
Culture of program evaluation (awareness, information)				0.566
**Component 2: Planning and implementation**				
Time and availability				-0.859
Technical assistance in economic evaluation				-0.773
Initial planning in the research protocol or implementation of HFPs				-0.661
Involvement in the decision-making process				-0.639
Planning and allocation of financial resources				-0.621
**Individual factors**	**N**	**KMO (Determinant)**	**Cumulative variance**	**Loadings** **after rotation**
	106	0.813 (0.082)	70.55%	
**Component 1: Training**				
Feeling unable to make a change				0.845
Educational level				0.820
Access to information				0.698
**Component 2: Motivation**				
Personal will				-0.943
Level of motivation				-0.809
Level of competence				-0.528
**Contextual factors**	**N**	**KMO (Determinant)**	**Cumulative variance**	**Loadings** **after rotation**
	106	0.801 (0.045)	72.22%	
**Component 1: Funding**				
Willingness of the funder during the funding process				0.857
Presence of a partnership and formal commitment				0.764
Support				0.684
**Component 2: Political economy**				
Economic level				0.787
Political dynamics				0.753
**Component 3: Public expectations**				
Collective expectation of the community				0.772
Predominance of the biomedical model (clinical effectiveness)				0.718
Current trends				0.637

*Kaiser—Meyer—Olkin.

For the organizational factors, we selected a two-component model that accounted for 61.9% of the variance. The first component, which was named ‘policy and governance,’ included the following five factors: the political will of the decision-maker, governance and priority of decision-makers, collaboration between the local administration and the regional and central administration, leadership, and the culture of program evaluation. The second component, which was named ‘planning and implementation,’ also included five factors: time and availability, technical assistance in EE, initial planning in the research protocol or implementation of HFPs, involvement in the decision-making process, and the planning and allocation of financial resources.

For the individual factors, a two-component model was chosen, accounting for 70.6% of the variance (Table 7). The first component, which we named ‘training,’ encompassed three factors: feeling unable to make a change, educational level, and access to information. The second component, which we named ‘motivation,’ also included three factors: personal will, level of motivation, and level of competence.

For the contextual factors, we chose a three-component model that accounted for 72.2% of the variance (Table 7). The first component, which was named ‘funding,’ included the following three factors: the willingness of the funder during the funding process, presence of a partnership and formal commitment, and support. The second component, which was named ‘political economy,’ encompassed economic level and political dynamics. The third component, which we named ‘public expectations,’ comprised the following three factors: collective expectation of the community, predominance of the biomedical model, and current trends.

## Discussion

We conducted a cross-sectional study to examine the knowledge, attitude, and practices of 106 stakeholders in relation to conducting an EE of Cameroonian HFPs. Of the 106 participants, 57.5% had heard about EEs; yet, nearly 90% reported that the HFP in question had never been subject to an EE nor was one planned. Furthermore, 84% of participants had never been involved in an EE of any of the HFPs. These findings point to the gap between the theoretical knowledge of Cameroonian stakeholders and practical application of their knowledge to the conception, development, and implementation of EEs of the existing HFPs. Low involvement of local stakeholders in the conception and development of EEs hinders improvements to the HFPs as well as identification of and investment into those that are most cost-effective [15]. This situation is not unique to Cameroon, but is experienced by many low- and middle-income countries (LMICs), which can be attributed to these countries’ limited capacity to conduct EEs, challenges with obtaining health economic data, and absence of a national research institute to provide the infrastructure necessary for conducting health EEs [30].

Most participants in our study expressed an intention or desire to be involved in EEs of the HFPs and receive proper training. Training in the form of a face-to-face seminar, workshop, or conference was preferred. These findings suggest both a lack of available training in conducting EEs in Cameroon and the need to invest in on-the-job training, mentoring, and continuous education of HCPs, managers, and policy makers within the Cameroonian healthcare system [31]. The required training should be delivered in an efficient and equitable manner to ensure that limited educational resources are allocated to Cameroonian organizations based on the priorities of a given segment of the population [32]. In addition, based on a learning needs assessment for implementation science training in LMICs conducted by Turner et al. [33], it can be recommended that training in EE in Cameroon be informed by stakeholders’ existing knowledge, delivered in an interactive format, adapted to the local context, and aimed at covering a variety of topics for a range of competencies.

PCAs identified organizational, individulal, and contextual factors that could influence the implementation of EEs of Cameroonian HFPs. Among the organizational factors, two components were identified: 1) policy and governance, and 2) planning and implementation. These results are consistent with a study that examined how 20 LMICs scored on multiple indicators (e.g. accountability, strategic vision) with respect to these countries’ governance of their healthcare workforce [34]. In the same study, barriers to good health system governance included restricted financial and human resource capacity, ineffective leadership, poor coordination between the public and private sectors, and a lack of a monitoring and evaluation plans. Among the individual factors, training and motivation emerged as principal components, which is in line with findings from a qualitative study of factors influencing health research capacity in Cameroon, Ethiopia, and Sri Lanka [35]. In this study, major barriers to participants’ involvement in health research included personal motivation, which was partly shaped by a lack of a conducive environment and financial and career incentives, as well as a shortage of skilled staff due to low prioritization of research methods in university curricula and continuing education. Among the contextual factors, the following components were established: 1) funding, 2) political economy, and 3) public expectations. Corroborating evidence comes from a political economy analysis of a performance-based financing program initiated in Afghanistan in 2010 [36]. This analysis uncovered characteristics that supported the program’s adoption, including positive examples of program implementation in other LMICs, the program’s alignment with donors’ interest in concrete results, and active involvement of local stakeholders in program design and implementation.

### Limitations

Our findings should be interpreted with caution due to several limitations. Primary stakeholders (i.e., beneficiaires of HFPs) were not included as it was beyond the scope of this study. Future research should include primary stakeholders to obtain a more complete picture of the state of HFPs in Cameroon. The cross-sectional design, small sample size, non-probability sampling methods, and non-inferential statistical analyses limit the generalizability of the results to other areas of Cameroon as well as other African countries. These limitations could be justified by the exploratory nature of this project and were partly addressed by interviewing a wide range of stakeholders involved in all of the 30 Cameroonian HFPs. In addition, the International Monetary Fund’s emergency response to COVID-19 fund disbursed to Cameroon in 2020 was not included in our study’s list of HFPs [37].

## Conclusion

Our findings underscore the importance of investing in the development of physical, human, material, and technological infrastructure necessary for performing EEs of Cameroonian HFPs as well as incentivising local stakeholders to make policy decisions based on the evidence generated by these evaluations [38]. Improved knowledge, diversified skills, and increased participation of stakeholders from all levels of the Cameroonian healthcare system are paramount to the effective and efficient development, implementation, and EE of the country’s existing HFPs.

## Supporting information

S1 Checklist Inclusivity in global research questionnaire. (DOCX)

S1 Questionnaire (DOCX)

S1 Table Participants’ knowledge of and level of involvement in Cameroon’s healthcare financing programs (N = 105). (DOCX)

S2 Table Participants’ level of involvement in a health economic evaluation of Cameroon’s healthcare financing programs (N = 106). (DOCX)

S3 Table Participants’ knowledge of whether Cameroon’s healthcare financing programs have been the subject of economic evaluation (N = 106). (DOCX)
