# Supplementary material for: Knowledge, attitude, and practices of stakeholders involved in healthcare financing programs on economic evaluations in Cameroon
Source: PLOS Glob Public Health. 2024 Apr 25;4(4):e0003101. doi: 10.1371/journal.pgph.0003101 (PMC11045103; doi:10.1371/journal.pgph.0003101)
Supplement: S1 Questionnaire — (DOCX) [file pgph.0003101.s002.docx]

**Questionnaire**

**English version**

**Assessment of the need for an economic evaluation of health financing policies by health personnel, managers, and policy makers in Cameroon**

Questionnaire to be administered to Ministry of Health staff, other ministry staff, hospital staff, health center staff, researchers, NGOs, international institutions to analyze needs for economic evaluation of health financing policies and programs

**Interviewer’s code: ________**

**Survey date: ____/_____/______/ (YY/MM/DD)**

**A. Identification**

A1. Sex: 1. Male 2. Female

A2. Type of organization: 1. Ministry of Health 2. Other Ministry 3. Hospital 4. Health centre 5. NGOs 6. International institutions

7. Academic institutions 8. Other: ________

A3. Name of organization: ________

A4. Professional title: ________

A5. Employment category: ________

A6. Place of residence: 1. Yaoundé 2. Douala 3. Bafoussam 4. Bameda

A7. Professional experience (years): ________

A8. How long have you worked in this organization/program (years)? ____

A9. Age (years) : _____

**B. Participants’ knowledge of and level of involvement in Cameroonian health financing programs**

| Health financing program | B1. Do you know this program? (check if ‘yes’) | B2. No involvement | B3. Level of Involvement | | | | | |
| --- | --- | --- | --- | --- | --- | --- | --- | --- |
|  |  |  | B3.1. Theoretical design | B3.2. Design in terms of consultation/  discussion with funders or funding policy | B3.3. Implementation in terms of program coordination or funding policy | B3.4. Implementation on the ground | B3.5. Evaluation | B3.6. Program improvement |
| **Free/subsidy policy focusing on disease control for the entire population** | | | | | | | | |
| Subsidized treatment for diabetes |  |  |  |  |  |  |  |  |
| Free care for epilepsy |  |  |  |  |  |  |  |  |
| Free care for preventive treatment of onchocerciasis |  |  |  |  |  |  |  |  |
| Free care for HIV/AIDS |  |  |  |  |  |  |  |  |
| Free treatment for tuberculosis |  |  |  |  |  |  |  |  |
| Free treatment for leprosy |  |  |  |  |  |  |  |  |
| Free treatment for Buruli ulcer |  |  |  |  |  |  |  |  |
| Subsidized treatment for cancer |  |  |  |  |  |  |  |  |
| **Free/subsidy policy focusing on controlling a disease targeting part of the population** | | | | | | | | |
| Free care for U5 malaria treatment |  |  |  |  |  |  |  |  |
| Subsidized malaria treatment for children over 5 years old and adults |  |  |  |  |  |  |  |  |
| Free intermittent preventing treatment (IPT) for pregnant women |  |  |  |  |  |  |  |  |
| Free long lasting insecticidal (LLI) bed nets |  |  |  |  |  |  |  |  |
| Free care for malnutrition |  |  |  |  |  |  |  |  |
| Free treatment for intestinal helminthiasis |  |  |  |  |  |  |  |  |
| Free treatment for schistosomiasis |  |  |  |  |  |  |  |  |
| Free care for diabetes (0-18 years) |  |  |  |  |  |  |  |  |
| **Free care on services** | | | | | | | | |
| Free care for family planning |  |  |  |  |  |  |  |  |
| **Free care for indigents** | | | | | | | | |
| Free care for abandoned children |  |  |  |  |  |  |  |  |
| Free care for indigents |  |  |  |  |  |  |  |  |
| **Budget financing** | | | | | | | | |
| Subvention for care in confessional facilities |  |  |  |  |  |  |  |  |
| Budget support for public health facilities |  |  |  |  |  |  |  |  |
| **Budget support targeting a segment of the population** | | | | | | | | |
| Medical evacuation funds (abroad) |  |  |  |  |  |  |  |  |
| Subsidized care for civil servants and health personnel |  |  |  |  |  |  |  |  |
| **Prepayment mechanism** | | | | | | | | |
| National health insurance |  |  |  |  |  |  |  |  |
| Social security |  |  |  |  |  |  |  |  |
| Private health insurance |  |  |  |  |  |  |  |  |
| Mutual health organization |  |  |  |  |  |  |  |  |
| **Results based financing** | | | | | | | | |
| Voucher |  |  |  |  |  |  |  |  |
| Performance based financing |  |  |  |  |  |  |  |  |
| **Payment at the point of service** | | | | | | | | |
| Out of pocket payment |  |  |  |  |  |  |  |  |
| **Other programs** | | | | | | | | |
| 1. |  |  |  |  |  |  |  |  |
| 2. |  |  |  |  |  |  |  |  |
| 3. |  |  |  |  |  |  |  |  |

**C. Participants’ knowledge of health economic evaluation**

C1. Have you ever heard of health economic evaluations? 1. Yes 2. No (if ‘no’, proceed to C9)

C2. If ‘yes’ to C1, what was the source of information (check all that apply)? 1. Health personnel 2. Community health worker 3. Media (radio/TV) 4. Internet 5. NGOs 6. Donors 7. University training 8. Training seminar/workshop 9. Conference 10. Other ___________

C3. In your opinion, what is the purpose of health economic evaluations (check all that apply)? 1. Generate cost estimates 2. Make decisions

3. Allocate resources 4. Produce economic reports 5. Know the target population 6. Have information on epidemics 7. Know the effectiveness of an intervention 8. Know the efficiency of an intervention 9. Don't know 10. Other ___________

C4. What types of health economic evaluations are you familiar with (check all that apply)? 1. None 2. Cost analysis 3. Cost-minimization analysis 4. Cost-effectiveness analysis 5. Cost-utility analysis 6. Cost-benefit analysis 7. Cost-consequences analysis 8. Other __________

C5. In your opinion, should your organization play a role in the implementation of health economic evaluations? 1. Yes 2. No

C6. If ‘yes’ to C5, what role?____________________________________________________

C7. In your opinion, should other organizations play a role in the implementation of health economic evaluations? 1. Yes 2. No

C8. If ‘yes’ to C7, please list these organizations and indicate each organization’s potential role in the implementation of health economic evaluations:

| Organizations | Organization 1: _____ | Organization 2: _____ | Organization 3: _____ | Organization 4: ______ | Organization 5: ____ |
| --- | --- | --- | --- | --- | --- |
| Roles |  |  |  |  |  |

**C9-C12. For each program, indicate whether it has already been subject to economic evaluation or whether an evaluation is/should be planned:**

| Health financing program | C9. Has already been the subject of economic analysis | C10. Don’t know if the program has ever been the subject of economic analysis or if it is planned | C11. Has never been the subject of economic analysis and it is not planned | C12. Has never been the subject of economic analysis, but it should be planned or carried out. |
| --- | --- | --- | --- | --- |
| **Free/subsidy policy focusing on disease control for the entire population** | | | | |
| Subsidized treatment for diabetes |  |  |  |  |
| Free care for epilepsy |  |  |  |  |
| Free care for preventive treatment of onchocerciasis |  |  |  |  |
| Free care for HIV/AIDS |  |  |  |  |
| Free treatment for tuberculosis |  |  |  |  |
| Free treatment for leprosy |  |  |  |  |
| Free treatment for Buruli ulcer |  |  |  |  |
| Subsidized treatment for cancer |  |  |  |  |
| **Free/subsidy policy focusing on controlling a disease targeting part of the population** | | | | |
| Free care for U5 malaria treatment |  |  |  |  |
| Subsidized malaria treatment for children over 5 years old and adults |  |  |  |  |
| Free intermittent preventing treatment (IPT) for pregnant women |  |  |  |  |
| Free long lasting insecticidal (LLI) bed nets |  |  |  |  |
| Free care for malnutrition |  |  |  |  |
| Free treatment for intestinal helminthiasis |  |  |  |  |
| Free treatment for schistosomiasis |  |  |  |  |
| Free care for diabetes (0-18 years) |  |  |  |  |
| **Free care on services** | | | | |
| Free care for family planning |  |  |  |  |
| **Free care for indigents** | | | | |
| Free care for abandoned children |  |  |  |  |
| Free care for indigents |  |  |  |  |
| **Budget financing** | | | | |
| Subvention for care in confessional facilities |  |  |  |  |
| Budget support for public health facilities |  |  |  |  |
| **Budget support targeting a segment of the population** | | | | |
| Medical evacuation funds (abroad) |  |  |  |  |
| Subsidized care for civil servants and health personnel |  |  |  |  |
| **Prepayment mechanism** | | | | |
| National health insurance |  |  |  |  |
| Social security |  |  |  |  |
| Private health insurance |  |  |  |  |
| Mutual health organization |  |  |  |  |
| **Results based financing** | | | | |
| Voucher |  |  |  |  |
| Performance based financing |  |  |  |  |
| **Payment at the point of service** | | | | |
| Out of pocket payment |  |  |  |  |
| **Other programs** | | | | |
| 1. |  |  |  |  |
| 2. |  |  |  |  |
| 3. |  |  |  |  |

**C13-C19. For each program that has already been subject to economic evaluation, specify the type of evaluation used:**

| Health financing program | Has already been the subject of… | | | |  |  |  |
| --- | --- | --- | --- | --- | --- | --- | --- |
|  | C13. Cost analysis | C14. Effects/  consequences analysis | C15. Cost- minimization analysis | C16. Cost-effectiveness analysis | C17. Cost-utility analysis | C18. Cost-benefit analysis | C19. Cost-consequences analysis |
| **Free/subsidy policy focusing on disease control for the entire population** | | | | | | | |
| Subsidized treatment for diabetes |  |  |  |  |  |  |  |
| Free care for epilepsy |  |  |  |  |  |  |  |
| Free care for preventive treatment of onchocerciasis |  |  |  |  |  |  |  |
| Free care for HIV/AIDS |  |  |  |  |  |  |  |
| Free treatment for tuberculosis |  |  |  |  |  |  |  |
| Free treatment for leprosy |  |  |  |  |  |  |  |
| Free treatment for Buruli ulcer |  |  |  |  |  |  |  |
| Subsidized treatment for cancer |  |  |  |  |  |  |  |
| **Free/subsidy policy focusing on controlling a disease targeting part of the population** | | | | | | | |
| Free care for U5 malaria treatment |  |  |  |  |  |  |  |
| Subsidized malaria treatment for children over 5 years old and adults |  |  |  |  |  |  |  |
| Free intermittent preventing treatment (IPT) for pregnant women |  |  |  |  |  |  |  |
| Free long lasting insecticidal (LLI) bed nets |  |  |  |  |  |  |  |
| Free care for malnutrition |  |  |  |  |  |  |  |
| Free treatment for intestinal helminthiasis |  |  |  |  |  |  |  |
| Free treatment for schistosomiasis |  |  |  |  |  |  |  |
| Free care for diabetes (0-18 years) |  |  |  |  |  |  |  |
| **Free care on services** | | | | | | | |
| Free care for family planning |  |  |  |  |  |  |  |
| **Free care for indigents** | | | | | | | |
| Free care for abandoned children |  |  |  |  |  |  |  |
| Free care for indigents |  |  |  |  |  |  |  |
| **Budget financing** | | | | | | | |
| Subvention for care in confessional facilities |  |  |  |  |  |  |  |
| Budget support for public health facilities |  |  |  |  |  |  |  |
| **Budget support targeting a segment of the population** | | | | | | | |
| Medical evacuation funds (abroad) |  |  |  |  |  |  |  |
| Subsidized care for civil servants and health personnel |  |  |  |  |  |  |  |
| **Prepayment mechanism** | | | | | | | |
| National health insurance |  |  |  |  |  |  |  |
| Social security |  |  |  |  |  |  |  |
| Private health insurance |  |  |  |  |  |  |  |
| Mutual health organization |  |  |  |  |  |  |  |
| **Results based financing** | | | | | | | |
| Voucher |  |  |  |  |  |  |  |
| Performance based financing |  |  |  |  |  |  |  |
| **Payment at the point of service** | | | | | | | |
| Out of pocket payment |  |  |  |  |  |  |  |
| **Other programs** | | | | | | | |
| 1. |  |  |  |  |  |  |  |
| 2. |  |  |  |  |  |  |  |
| 3. |  |  |  |  |  |  |  |

C20. Do you think that the programs you mentioned that have not yet been subject to economic evaluation should undergo an evaluation?

1. Yes 2. No

**D. Participation in / practice of health economic evaluation**

D1. Has someone been assigned to perform economic evaluations in your organization? 1. Yes 2. No

D2. If ‘yes’ to D1, do you know if this person has performed economic evaluations? 1. Yes 2. No

D3. Have you participated in a health economic evaluation? 1. Yes 2. No

D4. If ‘yes’ to D3, have you ever been involved in an economic evaluation of the following programs, and if yes, what was your level of involvement?

| Health financing program | D4 .1 No involvement | D4.2. Level of involvement in a health economic evaluation: | | | | | |
| --- | --- | --- | --- | --- | --- | --- | --- |
|  |  | D4.2.1. Initial and theoretical design | D4.2.2. Design in terms of consultation/  discussion with donors | D4.2.3. Implementation in terms of coordination | D4.2.4. Implementation on the ground | D4.2.5. Data analysis | D4.2.6. Improvement |
| **Free/subsidy policy focusing on disease control for the entire population** | | | | | | | |
| Subsidized treatment for diabetes |  |  |  |  |  |  |  |
| Free care for epilepsy |  |  |  |  |  |  |  |
| Free care for preventive treatment of onchocerciasis |  |  |  |  |  |  |  |
| Free care for HIV/AIDS |  |  |  |  |  |  |  |
| Free treatment for tuberculosis |  |  |  |  |  |  |  |
| Free treatment for leprosy |  |  |  |  |  |  |  |
| Free treatment for Buruli ulcer |  |  |  |  |  |  |  |
| Subsidized treatment for cancer |  |  |  |  |  |  |  |
| **Free/subsidy policy focusing on controlling a disease targeting part of the population** | | | | | | | |
| Free care for U5 malaria treatment |  |  |  |  |  |  |  |
| Subsidized malaria treatment for children over 5 years old and adults |  |  |  |  |  |  |  |
| Free intermittent preventing treatment (IPT) for pregnant women |  |  |  |  |  |  |  |
| Free long lasting insecticidal (LLI) bed nets |  |  |  |  |  |  |  |
| Free care for malnutrition |  |  |  |  |  |  |  |
| Free treatment for intestinal helminthiasis |  |  |  |  |  |  |  |
| Free treatment for schistosomiasis |  |  |  |  |  |  |  |
| Free care for diabetes (0-18 years) |  |  |  |  |  |  |  |
| **Free care on services** | | | | | | | |
| Free care for family planning |  |  |  |  |  |  |  |
| **Free care for indigents** | | | | | | | |
| Free care for abandoned children |  |  |  |  |  |  |  |
| Free care for indigents |  |  |  |  |  |  |  |
| **Budget financing** | | | | | | | |
| Subvention for care in confessional facilities |  |  |  |  |  |  |  |
| Budget support for public health facilities |  |  |  |  |  |  |  |
| **Budget support targeting a segment of the population** | | | | | | | |
| Medical evacuation funds (abroad) |  |  |  |  |  |  |  |
| Subsidized care for civil servants and health personnel |  |  |  |  |  |  |  |
| **Prepayment mechanism** | | | | | | | |
| National health insurance |  |  |  |  |  |  |  |
| Social security |  |  |  |  |  |  |  |
| Private health insurance |  |  |  |  |  |  |  |
| Mutual health organization |  |  |  |  |  |  |  |
| **Results based financing** | | | | | | | |
| Voucher |  |  |  |  |  |  |  |
| Performance based financing |  |  |  |  |  |  |  |
| **Payment at the point of service** | | | | | | | |
| Out of pocket payment |  |  |  |  |  |  |  |
| **Other programs** | | | | | | | |
| 1. |  |  |  |  |  |  |  |
| 2. |  |  |  |  |  |  |  |
| 3. |  |  |  |  |  |  |  |

**E. Attitudes toward economic evaluations of health financing policies and programs**

E1. Do you intend to or would you like to be involved in the theoretical design of an economic evaluation of a health financing program or policy?

1. Yes 2. No

E2. Do you intend to or would you like to be involved in the design in terms of consultation or discussion with funders for an economic evaluation of a financing program or policy? 1. Yes 2. No

E3. Do you intend or would you like to be involved in the implementation in terms of coordination of an economic evaluation of a health financing program or policy? 1. Yes 2. No

E4. Do you intend to or would you like to be involved in the implementation on the ground of an economic evaluation of a health financing program or policy? 1. Yes 2. No

E5. Do you intend to or would you like to be involved in the data analysis for an economic evaluation of a health financing program or policy?

1. Yes 2. No

E6. Do you intend to or would you like to be involved in the improvement of a health financing program or policy based on the results of an economic evaluation? 1. Yes 2. No

E7. Would you like or do you intend to receive training in health economic evaluations? 1. Yes 2. No

E8. If ‘yes’ to E7, which form of training would you prefer to receive (check all that apply)? 1. Face-to-face seminar/workshop 2. Face-to-face conference 3. Online seminar/workshop 4. Online conference 5. Online training capsule

**F. Factors influencing participants’ involvement in the economic evaluation of health financing programs and policies**

What is your level of agreement for the following factors in terms of their potential to influence your involvement in the economic analysis of healthcare financing programs and policies?

*1. Strongly disagree 2. Disagree 3. Slightly agree 4. Agree 5. Strongly agree*

| **Factors** | 1 | 2 | 3 | 4 | 5 |
| --- | --- | --- | --- | --- | --- |
| **Institutional factors** | | | | | |
| Political will of the decision-maker |  |  |  |  |  |
| Governance and priority of decision-makers |  |  |  |  |  |
| Culture of program evaluation (awareness, information) |  |  |  |  |  |
| Planning and allocation of financial resources |  |  |  |  |  |
| Leadership |  |  |  |  |  |
| Involvement in the decision-making process |  |  |  |  |  |
| Technical assistance in economic evaluation |  |  |  |  |  |
| Time and availability |  |  |  |  |  |
| Initial planning in the research protocol or implementation of health financing policies and programs |  |  |  |  |  |
| Collaboration between the local and regional/central administration |  |  |  |  |  |
| **Individual factors** | | | | | |
| Access to information |  |  |  |  |  |
| Level of training |  |  |  |  |  |
| Level of competence |  |  |  |  |  |
| Feeling unable to make a change |  |  |  |  |  |
| Level of motivation |  |  |  |  |  |
| Personal will |  |  |  |  |  |
| **Contextual factors** | | | | | |
| Political dynamics |  |  |  |  |  |
| Economic level |  |  |  |  |  |
| Presence of a partnership and formal commitment |  |  |  |  |  |
| Willingness of the funder during the funding process |  |  |  |  |  |
| Support |  |  |  |  |  |
| Predominance of the biomedical model (clinical effectiveness) |  |  |  |  |  |
| Availability of a regulatory and legal framework |  |  |  |  |  |
| Advocacy |  |  |  |  |  |
| Collective expectation of the community |  |  |  |  |  |
| Current trends |  |  |  |  |  |
| Access to technology |  |  |  |  |  |
| **Other factors** | | | | | |
| 1. |  |  |  |  |  |
| 2. |  |  |  |  |  |
| 3. |  |  |  |  |  |

**French version**

**Évaluation des besoins en analyse économique des politiques de financement de la santé par le personnel de santé, les gestionnaires et les décideurs politiques au Cameroun**

Questionnaire à administrer au personnel du ministère de la santé, au personnel autres ministère, au personnel des hôpitaux, au personnel du centre de santé, chercheurs, ONG, institutions internationales pour analyser les besoins en analyse économique des politiques et programmes de financement de la santé

**Code de l’agent enquêteur ou l’agente enquêtrice: ________**

**Date de l’enquête : ____/_____/______/ (JJ/MM/AA)**

**A. Identification**

A1. Sexe : 1. Homme 2. Femme

A2. Type de structure : 1. Ministère de la santé 2. Autre ministère 3. Hôpital 4. Centre de santé 5. ONG/associations 6. Institutions internationales 7. Institutions universitaires 8. Autre : ________

A3. Nom de la structure : ________

A4. Poste occupé : ________

A5. Catégorie : ________

A6. Ville d’appartenance : 1. Yaoundé 2. Douala 3. Bafoussam 4. Bameda

A7. Expérience professionnelle (en années): ________

A8. Depuis combien de temps (en années) travaillez-vous dans cette structure/programme? ____

A9. Âge : _____

**B. Connaissance et implication dans les politiques et programmes de financement de la santé au Cameroun**

| Politiques et programmes de financement existants | B1. Connaissez-vous ce programme ou politique (cochez si oui) | B2. Aucune implication | B3. Degré d’implication | | | | | |
| --- | --- | --- | --- | --- | --- | --- | --- | --- |
|  |  |  | B3.1. La conception initiale et théorique | B3.2. La conception en termes de consultation ou discussion avec les bailleurs de fonds | B3.3. La mise en œuvre en terme de coordination | B3.4. La mise en œuvre terrain | B3.5. L’évaluation | B3.6. Le  réajustement |
| **Politique de gratuité / subvention axée sur la lutte contre la maladie pour l'ensemble de la population** | | | | | | | | |
| Traitement subventionné du diabète |  |  |  |  |  |  |  |  |
| Épilepsie de soins gratuits |  |  |  |  |  |  |  |  |
| Soins gratuits pour le traitement préventif de l’onchocercose |  |  |  |  |  |  |  |  |
| Prise en charge gratuite du VIH / SIDA |  |  |  |  |  |  |  |  |
| Prise en charge gratuite de la tuberculose |  |  |  |  |  |  |  |  |
| Soins gratuits pour la lèpre |  |  |  |  |  |  |  |  |
| Soins gratuits pour l'ulcère de Buruli |  |  |  |  |  |  |  |  |
| Traitement subventionné du cancer |  |  |  |  |  |  |  |  |
| **Politique de gratuité / subvention axée sur le contrôle d'une maladie ciblant une partie de la population** | | | | | | | | |
| Soins gratuits pour le traitement du paludisme U5 |  |  |  |  |  |  |  |  |
| Traitement antipaludique subventionné pour les enfants de plus de 5 ans et les adultes |  |  |  |  |  |  |  |  |
| IPT gratuit pour les femmes enceintes |  |  |  |  |  |  |  |  |
| Filets de lit LLI gratuits |  |  |  |  |  |  |  |  |
| Prise en charge gratuite de la malnutrition |  |  |  |  |  |  |  |  |
| Traitement gratuit des helminthiases intestinales |  |  |  |  |  |  |  |  |
| Prise en charge gratuite de la schistosomiase |  |  |  |  |  |  |  |  |
| Soins gratuits pour le diabète de 0 à 18 ans |  |  |  |  |  |  |  |  |
| **Soins gratuits sur les services** | | | | | | | | |
| Prise en charge gratuite de la planification familiale |  |  |  |  |  |  |  |  |
| **Soins gratuits pour les indigents** | | | | | | | | |
| Prise en charge gratuite des enfants abandonnés |  |  |  |  |  |  |  |  |
| Soins gratuits pour les indigents |  |  |  |  |  |  |  |  |
| **Financement budgétaire** | | | | | | | | |
| Subvention pour soins dans les établissements confessionnels |  |  |  |  |  |  |  |  |
| Appui budgétaire aux établissements de santé publique |  |  |  |  |  |  |  |  |
| **Aide budgétaire ciblant un segment de la population** | | | | | | | | |
| Fonds d'évacuation sanitaire (à l'étranger) |  |  |  |  |  |  |  |  |
| Soins subventionnés pour les fonctionnaires et le personnel de santé |  |  |  |  |  |  |  |  |
| **Mécanisme de prépaiement** | | | | | | | | |
| Assurance maladie nationale |  |  |  |  |  |  |  |  |
| Sécurité sociale |  |  |  |  |  |  |  |  |
| Assurance maladie privée |  |  |  |  |  |  |  |  |
| Organisation de mutuelles de santé |  |  |  |  |  |  |  |  |
| **Financement basé sur les résultats** | | | | | | | | |
| Chèque santé (Voucher) |  |  |  |  |  |  |  |  |
| Financement basé sur la performance |  |  |  |  |  |  |  |  |
| **Paiement au point de service** | | | | | | | | |
| Paiement direct |  |  |  |  |  |  |  |  |
| **Autres programmes** | | | | | | | | |
| 1. |  |  |  |  |  |  |  |  |
| 2. |  |  |  |  |  |  |  |  |
| 3. |  |  |  |  |  |  |  |  |

**C. Connaissances de l’évaluation économique en santé par la personne répondante**

C1. Avez-vous déjà entendu parler de l’évaluation économique en santé? 1. Oui 2. Non (*Sinon allez à C9)*

C2. Si oui à C1, quelle est la source de votre information (plusieurs choix)? 1. Personnel de santé 2. Agent de santé communautaire 3. Médias (radio/télévision) 4. Internet 5. ONG 6. Bailleurs de fonds 7. Formation universitaire 8. Séminaire et atelier de formation 9. Conférences 10. Autre ______________

C3. Selon vous, à quoi sert l’évaluation économique en santé (plusieurs choix)? 1. Produire des estimations de coûts 2. Prise de décision 3. Allocation des ressources 4. Produire des rapports et argumentaires d’ordre économique 5. Connaitre la population desservie 6. Avoir des informations sur les épidémies 7. Connaitre l’efficacité d’une intervention 8. Connaitre l’efficience d’une intervention 9. Ne sait pas 10. Autre ______________

C4. Quelles sont les différentes approches d’évaluation économique en santé que vous connaissez (plusieurs choix)? 1. Aucune 2. Analyse des coûts 3. Analyse de minimisation de coûts 4. Analyse coût efficacité 5. Analyse coût utilité 6. Analyse coût bénéfice 7. Coût conséquence 8. Autre ______________

C5. Selon vous, votre structure devrait-elle jouer un rôle dans la mise en œuvre de l’évaluation économique en santé?

1. Oui 2. Non

C6. Si oui C5, lequel?____________________________________________________

C7. Selon vous, d’autres structures devraient-elles jouer un rôle dans les évaluations économiques en santé? 1. Oui 2. Non

C8. Si oui à C7, lister ces structures et indiquer pour chacune d’elles leur rôle potentiel dans la connaissance des évaluations économiques :

| Structures | Structure 1 : _____ | Structure 2 : _____ | Structure 3 : _____ | Structure 4 : ______ | Structure 5 : _____ |
| --- | --- | --- | --- | --- | --- |
| Rôles joués |  |  |  |  |  |

**C9-C12. Pour chacun des programmes de financement, indiquez s’il a bénéficié d’une évaluation économique et si elle est planifiée ou devrait être planifiée :**

| Politiques et programmes de financement existants | C9. Ce programme de financement a déjà fait l’objet d’une analyse économique | C10. Je ne sais pas si elle a déjà fait l’objet d’une analyse économique ou si une est planifiée | C11. N'a jamais fait l'objet d'une analyse économique, et elle n’est pas planifiée | C12. N'a jamais fait l'objet d'une analyse économique, mais devrait être planifiée ou réalisée |
| --- | --- | --- | --- | --- |
| **Politique de gratuité / subvention axée sur la lutte contre la maladie pour l'ensemble de la population** | | | | |
| Traitement subventionné du diabète |  |  |  |  |
| Épilepsie de soins gratuits |  |  |  |  |
| Soins gratuits pour le traitement préventif de l’onchocercose |  |  |  |  |
| Prise en charge gratuite du VIH / SIDA |  |  |  |  |
| Prise en charge gratuite de la tuberculose |  |  |  |  |
| Soins gratuits pour la lèpre |  |  |  |  |
| Soins gratuits pour l'ulcère de Buruli |  |  |  |  |
| Traitement subventionné du cancer |  |  |  |  |
| **Politique de gratuité / subvention axée sur le contrôle d'une maladie ciblant une partie de la population** | | | | |
| Soins gratuits pour le traitement du paludisme U5 |  |  |  |  |
| Traitement antipaludique subventionné pour les enfants de plus de 5 ans et les adultes |  |  |  |  |
| IPT gratuit pour les femmes enceintes |  |  |  |  |
| Filets de lit LLI gratuits |  |  |  |  |
| Prise en charge gratuite de la malnutrition |  |  |  |  |
| Traitement gratuit des helminthiases intestinales |  |  |  |  |
| Prise en charge gratuite de la schistosomiase |  |  |  |  |
| Soins gratuits pour le diabète de 0 à 18 ans |  |  |  |  |
| **Soins gratuits sur les services** | | | | |
| Prise en charge gratuite de la planification familiale |  |  |  |  |
| **Soins gratuits pour les indigents** | | | | |
| Prise en charge gratuite des enfants abandonnés |  |  |  |  |
| Soins gratuits pour les indigents |  |  |  |  |
| **Financement budgétaire** | | | | |
| Subvention pour soins dans les établissements confessionnels |  |  |  |  |
| Appui budgétaire aux établissements de santé publique |  |  |  |  |
| **Aide budgétaire ciblant un segment de la population** | | | | |
| Fonds d'évacuation sanitaire (à l'étranger) |  |  |  |  |
| Soins subventionnés pour les fonctionnaires et le personnel de santé |  |  |  |  |
| **Mécanisme de prépaiement** | | | | |
| Assurance maladie nationale |  |  |  |  |
| Sécurité sociale |  |  |  |  |
| Assurance maladie privée |  |  |  |  |
| Organisation de mutuelles de santé |  |  |  |  |
| **Financement basé sur les résultats** | | | | |
| Chèque santé (Voucher) |  |  |  |  |
| Financement basé sur la performance |  |  |  |  |
| **Paiement au point de service** | | | | |
| Paiement direct |  |  |  |  |
| **Autres programmes** | | | | |
| 1. |  |  |  |  |
| 2. |  |  |  |  |
| 3. |  |  |  |  |

**C13-C19. Pour chacun des programmes de financement** **ayant bénéficié d’une évaluation économique, précisez le type d’approche utilisé :**

| Politiques et programmes de financement existants | A déjà fait l’objet d’une analyse… | | | | | | |
| --- | --- | --- | --- | --- | --- | --- | --- |
|  | C13. des coûts | C14. des effets/  conséquences | C15. de minimisation des coûts | C16. coûts-efficacité | C17. coûts-utilité | C18. coûts-bénéfice | C19. coûts-conséquence |
| **Politique de gratuité / subvention axée sur la lutte contre la maladie pour l'ensemble de la population** | | | | | | | |
| Traitement subventionné du diabète |  |  |  |  |  |  |  |
| Épilepsie de soins gratuits |  |  |  |  |  |  |  |
| Soins gratuits pour le traitement préventif de l’onchocercose |  |  |  |  |  |  |  |
| Prise en charge gratuite du VIH / SIDA |  |  |  |  |  |  |  |
| Prise en charge gratuite de la tuberculose |  |  |  |  |  |  |  |
| Soins gratuits pour la lèpre |  |  |  |  |  |  |  |
| Soins gratuits pour l'ulcère de Buruli |  |  |  |  |  |  |  |
| Traitement subventionné du cancer |  |  |  |  |  |  |  |
| **Politique de gratuité / subvention axée sur le contrôle d'une maladie ciblant une partie de la population** | | | | | | | |
| Soins gratuits pour le traitement du paludisme U5 |  |  |  |  |  |  |  |
| Traitement antipaludique subventionné pour les enfants de plus de 5 ans et les adultes |  |  |  |  |  |  |  |
| IPT gratuit pour les femmes enceintes |  |  |  |  |  |  |  |
| Filets de lit LLI gratuits |  |  |  |  |  |  |  |
| Prise en charge gratuite de la malnutrition |  |  |  |  |  |  |  |
| Traitement gratuit des helminthiases intestinales |  |  |  |  |  |  |  |
| Prise en charge gratuite de la schistosomiase |  |  |  |  |  |  |  |
| Soins gratuits pour le diabète de 0 à 18 ans |  |  |  |  |  |  |  |
| **Soins gratuits sur les services** | | | | | | | |
| Prise en charge gratuite de la planification familiale |  |  |  |  |  |  |  |
| **Soins gratuits pour les indigents** | | | | | | | |
| Prise en charge gratuite des enfants abandonnés |  |  |  |  |  |  |  |
| Soins gratuits pour les indigents |  |  |  |  |  |  |  |
| **Financement budgétaire** | | | | | | | |
| Subvention pour soins dans les établissements confessionnels |  |  |  |  |  |  |  |
| Appui budgétaire aux établissements de santé publique |  |  |  |  |  |  |  |
| **Aide budgétaire ciblant un segment de la population** | | | | | | | |
| Fonds d'évacuation sanitaire (à l'étranger) |  |  |  |  |  |  |  |
| Soins subventionnés pour les fonctionnaires et le personnel de santé |  |  |  |  |  |  |  |
| **Mécanisme de prépaiement** | | | | | | | |
| Assurance maladie nationale |  |  |  |  |  |  |  |
| Sécurité sociale |  |  |  |  |  |  |  |
| Assurance maladie privée |  |  |  |  |  |  |  |
| Organisation de mutuelles de santé |  |  |  |  |  |  |  |
| **Financement basé sur les résultats** | | | | | | | |
| Chèque santé (Voucher) |  |  |  |  |  |  |  |
| Financement basé sur la performance |  |  |  |  |  |  |  |
| **Paiement au point de service** | | | | | | | |
| Paiement direct |  |  |  |  |  |  |  |
| **Autres programmes** | | | | | | | |
| 1. |  |  |  |  |  |  |  |
| 2. |  |  |  |  |  |  |  |
| 3. |  |  |  |  |  |  |  |

C20. Pensez-vous que les programmes de financement de la santé que vous avez cité et qui n’ont pas fait l’objet d’évaluation économique devraient-ils aussi bénéficier d’une évaluation économique? 1. Oui 2. Non

**D. Participation / pratique de l’évaluation économique en santé**

D1. Y a-t-il une personne attitrée à l’évaluation dans votre structure? 1. Oui 2. Non

D2. Si oui à D1, savez-vous si cette personne fait de l’évaluation économique? 1. Oui 2. Non

D3. Avez-vous déjà participé à une évaluation économique en santé?

D4. Si oui à D3, avez-vous déjà participé à une évaluation économique d’un programme ou politique de la santé suivant, et quel a été votre niveau d’implication dans cette évaluation :

| Politiques et programmes de financement existants | D4.1. Aucune implication | D4.2. Implication dans l’analyse économique : | | | | | |
| --- | --- | --- | --- | --- | --- | --- | --- |
|  |  | D4.2.1. La conception initiale et théorique | D4.2.2. La conception en termes de consultation ou discussion avec les bailleurs de fonds | D4.2.3. La mise en œuvre en terme de coordination | D4.2.4. La mise en œuvre terrain | D4.2.5. L’analyse des données | D4.2.6. Le réajustement du programme/  politique à partir des résultats d’une évaluation |
| **Politique de gratuité / subvention axée sur la lutte contre la maladie pour l'ensemble de la population** | | | | | | | |
| Traitement subventionné du diabète |  |  |  |  |  |  |  |
| Épilepsie de soins gratuits |  |  |  |  |  |  |  |
| Soins gratuits pour le traitement préventif de l’onchocercose |  |  |  |  |  |  |  |
| Prise en charge gratuite du VIH / SIDA |  |  |  |  |  |  |  |
| Prise en charge gratuite de la tuberculose |  |  |  |  |  |  |  |
| Soins gratuits pour la lèpre |  |  |  |  |  |  |  |
| Soins gratuits pour l'ulcère de Buruli |  |  |  |  |  |  |  |
| Traitement subventionné du cancer |  |  |  |  |  |  |  |
| **Politique de gratuité / subvention axée sur le contrôle d'une maladie ciblant une partie de la population** | | | | | | | |
| Soins gratuits pour le traitement du paludisme U5 |  |  |  |  |  |  |  |
| Traitement antipaludique subventionné pour les enfants de plus de 5 ans et les adultes |  |  |  |  |  |  |  |
| IPT gratuit pour les femmes enceintes |  |  |  |  |  |  |  |
| Filets de lit LLI gratuits |  |  |  |  |  |  |  |
| Prise en charge gratuite de la malnutrition |  |  |  |  |  |  |  |
| Traitement gratuit des helminthiases intestinales |  |  |  |  |  |  |  |
| Prise en charge gratuite de la schistosomiase |  |  |  |  |  |  |  |
| Soins gratuits pour le diabète de 0 à 18 ans |  |  |  |  |  |  |  |
| **Soins gratuits sur les services** | | | | | | | |
| Prise en charge gratuite de la planification familiale |  |  |  |  |  |  |  |
| **Soins gratuits pour les indigents** | | | | | | | |
| Prise en charge gratuite des enfants abandonnés |  |  |  |  |  |  |  |
| Soins gratuits pour les indigents |  |  |  |  |  |  |  |
| **Financement budgétaire** | | | | | | | |
| Subvention pour soins dans les établissements confessionnels |  |  |  |  |  |  |  |
| Appui budgétaire aux établissements de santé publique |  |  |  |  |  |  |  |
| **Aide budgétaire ciblant un segment de la population** | | | | | | | |
| Fonds d'évacuation sanitaire (à l'étranger) |  |  |  |  |  |  |  |
| Soins subventionnés pour les fonctionnaires et le personnel de santé |  |  |  |  |  |  |  |
| **Mécanisme de prépaiement** | | | | | | | |
| Assurance maladie nationale |  |  |  |  |  |  |  |
| Sécurité sociale |  |  |  |  |  |  |  |
| Assurance maladie privée |  |  |  |  |  |  |  |
| Organisation de mutuelles de santé |  |  |  |  |  |  |  |
| **Financement basé sur les résultats** | | | | | | | |
| Chèque santé (Voucher) |  |  |  |  |  |  |  |
| Financement basé sur la performance |  |  |  |  |  |  |  |
| **Paiement au point de service** | | | | | | | |
| Paiement direct |  |  |  |  |  |  |  |
| **Autres programmes** | | | | | | | |
| 1. |  |  |  |  |  |  |  |
| 2. |  |  |  |  |  |  |  |
| 3. |  |  |  |  |  |  |  |

**E. Attitudes face à l’évaluation économique des politiques et programmes de financement de la santé**

E1. Aviez-vous l’intention ou aimeriez-vous participer à la conception théorique d’une évaluation économique d’un programme ou d’une politique de financement de santé? 1 Oui 2. Non

E2. Aviez-vous l’intention ou aimeriez-vous participer à la conception en termes de consultation ou discussion avec les bailleurs de fonds pour l’évaluation économique du programme ou politique de financement? 1 Oui 2. Non

E3. Aviez-vous l’intention ou aimeriez-vous participer à la mise en œuvre en termes de coordination d’une évaluation économique du programme ou politique de financement de santé? 1 Oui 2. Non

E4. Aviez-vous l’intention ou aimeriez-vous participer Implication dans la mise en œuvre terrain de l’évaluation économique du programme ou politique de financement de santé? 1 Oui 2. Non

E5. Aviez-vous l’intention ou aimeriez-vous participer à Implication dans l’analyse des données d’une de l’évaluation économique du programme ou politique de financement de santé? 1 Oui 2. Non

E6. Aviez-vous l’intention ou aimeriez-vous participer au réajustement du programme ou politique de financement de santé à partir des résultats d’une évaluation économique? 1 Oui 2. Non

E7. Aimeriez-vous ou aviez-vous l’intention de participer à une formation en évaluation économique en santé? 1 Oui 2. Non

E8. Si oui à E7, quelle forme de formation préfériez-vous (plusieurs choix)? 1. Formation en présentiel sous forme de séminaire / atelier de travail 2. Formation en présentiel sous forme de conférence 3. Formation en ligne sous forme de séminaire / atelier de travail 4. Formation en ligne sous forme de conférence 5. Formation en ligne sous forme de capsule de formation

**F. Facteurs d’influencent de la participation des personnes répondantes dans l’analyse économique des programmes et politiques de financement de la santé**

Quelle est votre appréciation des affirmations suivantes en lien avec les facteurs qui pourraient influencer votre participation à l’analyse économique des programmes et politiques de financement de la santé?

*1. Fortement en désaccord 2. En désaccord 3. Peu en accord 4. En accord 5. Fortement en accord*

| **Facteurs** | 1 | 2 | 3 | 4 | 5 |
| --- | --- | --- | --- | --- | --- |
| **Facteurs institutionnels** | | | | | |
| La volonté politique du décideur |  |  |  |  |  |
| La gouvernance et priorité des décideurs |  |  |  |  |  |
| La culture de l’évaluation des projets et programmes (sensibilisation, information) |  |  |  |  |  |
| La planification et la répartition des ressources financières |  |  |  |  |  |
| Le leadership |  |  |  |  |  |
| L’implication dans le processus de prise de décision |  |  |  |  |  |
| L'assistance technique en évaluation économique |  |  |  |  |  |
| Le temps et la disponibilité |  |  |  |  |  |
| La planification initiale dans le protocole de recherche ou dans la mise en œuvre des politiques et programmes de financement de la santé |  |  |  |  |  |
| La collaboration entre le local et l'administration politique régionale et centrale |  |  |  |  |  |
| **Facteurs individuels** | | | | | |
| L'accès à l'information |  |  |  |  |  |
| Le niveau de formation |  |  |  |  |  |
| Le niveau de compétence |  |  |  |  |  |
| Le sentiment d'incapacité d'exercer un changement |  |  |  |  |  |
| Le niveau de motivation |  |  |  |  |  |
| La volonté personnelle |  |  |  |  |  |
| **Facteurs contextuels** | | | | | |
| La dynamique politique |  |  |  |  |  |
| Le niveau économique |  |  |  |  |  |
| La présence d'un partenariat et un engagement formelle |  |  |  |  |  |
| La volonté du bailleur de fonds lors du processus d’octroi des fonds |  |  |  |  |  |
| L'accompagnement |  |  |  |  |  |
| La prédominance du modèle biomédical (volet efficacité clinique) |  |  |  |  |  |
| La disponibilité d'un cadre règlementaire et juridique |  |  |  |  |  |
| La présence d'un plaidoyer |  |  |  |  |  |
| L'attente collective de la communauté |  |  |  |  |  |
| L’effet de mode ou mouvance actuelle |  |  |  |  |  |
| L'accès à la technologie |  |  |  |  |  |
| **Autres facteurs** | | | | | |
| 1. |  |  |  |  |  |
| 2. |  |  |  |  |  |
| 3. |  |  |  |  |  |
