# Supplementary material for: Knowledge, attitude, and practices of stakeholders involved in healthcare financing programs on economic evaluations in Cameroon
Source: PLOS Glob Public Health. 2024 Apr 25;4(4):e0003101. doi: 10.1371/journal.pgph.0003101 (PMC11045103; doi:10.1371/journal.pgph.0003101)
Supplement: S1 Table — (DOCX) [file pgph.0003101.s003.docx]

Participants’ knowledge of and level of involvement in Cameroon’s healthcare financing programs (N=105)

| **Healthcare financing programs** | **Do you know this program?** n (% selected) | **Some level of involvement** – n (% selected out of those who know the program) | **Level of involvement** – n (% selected out of those who indicated some level of involvement) | | | | | |
| --- | --- | --- | --- | --- | --- | --- | --- | --- |
|  |  |  | Theoretical design | Design in terms of consultation/discussion with funders or funding policy | Implementation in terms of program coordination or funding policy | Implementation on the ground | Evaluation | Program improvement |
| **Free/subsidy policy focusing on disease control for the entire population (8 programs)** | | | | | | | | |
| Subsidized treatment for diabetes | 50 (47.6) | 15 (30.0) | 0 (0.0) | 0 (0.0) | 0 (0.0) | 11 (73.3) | 1 (6.7) | 0 (0.0) |
| Free care for epilepsy | 38 (36.2) | 9 (23.7) | 0 (0.0) | 0 (0.0) | 0 (0.0) | 2 (22.2) | 1 (11.1) | 0 (0.0) |
| Free care for preventive treatment of onchocerciasis | 72 (68.6) | 23 (31.9) | 1 (4.3) | 1 (4.3) | 3 (13.0) | 15 (65.2) | 8 (34.8) | 3 (13.0) |
| Free care for HIV/AIDS | 97 (92.4) | 46 (47.4) | 6 (13.0) | 7 (15.2) | 8 (17.4) | 39 (84.8) | 19 (41.3) | 12 (26.1) |
| Free treatment for tuberculosis | 90 (85.7) | 31 (34.4) | 0 (0.0) | 2 (6.5) | 2 (6.5) | 25 (80.6) | 10 (32.3) | 3 (9.7) |
| Free treatment for leprosy | 60 (57.1) | 12 (20.0) | 1 (8.3) | 1 (8.3) | 2 (16.7) | 5 (41.7) | 3 (25.0) | 1 (8.3) |
| Free treatment for Buruli ulcer | 55 (52.4) | 15 (27.3) | 1 (6.7) | 2 (13.3) | 3 (20.0) | 6 (40.0) | 4 (26.7) | 2 (13.3) |
| Subsidized treatment for cancer | 48 (45.7) | 11 (22.9) | 0 (0.0) | 0 (0.0) | 1 (9.1) | 3 (27.3) | 1 (9.1) | 0 (0.0) |
| **Category Mean** | **63.8 (60.7)** | **20.3 (40.5)** | **1.1 (4.0)** | **1.6 (6.0)** | **2.4 (10.3)** | **13.3 (54.4)** | **5.9 (23.4)** | **2.6 (8.8)** |
| **Free/subsidy policy focusing on controlling a disease targeting part of the population (8 programs)** | | | | | | | | |
| Free malaria treatment for children under 5 years old | 96 (91.4) | 41 (42.7) | 4 (9.8) | 6 (14.6) | 10 (24.4) | 36 (87.8) | 19 (46.3) | 8 (19.5) |
| Subsidized malaria treatment for children over 5 years old and adults | 74 (70.5) | 36 (48.6) | 2 (5.6) | 4 (11.1) | 6 (16.7) | 29 (80.6) | 10 (27.8) | 4 (11.1) |
| Free intermittent preventing treatment (IPT) for pregnant women | 93 (88.6) | 38 (40.9) | 3 (7.9) | 5 (13.2) | 7 (18.4) | 31 (81.6) | 12 (31.6) | 5 (13.2) |
| Free long lasting insecticidal (LLI) bed nets | 87 (82.9) | 19 (21.8) | 3 (15.8) | 6 (31.6) | 6 (31.6) | 12 (63.2) | 9 (47.4) | 6 (31.6) |
| Free care for malnutrition | 65 (61.9) | 23 (35.4) | 1 (4.3) | 1 (4.3) | 3 (13.0) | 18 (78.3) | 6 (26.1) | 1 (4.3) |
| Free treatment for intestinal helminthiasis | 72 (68.6) | 22 (30.6) | 1 (4.5) | 4 (18.2) | 2 (9.1) | 14 (63.6) | 5 (22.7) | 4 (18.2) |
| Free treatment for schistosomiasis | 56 (53.3) | 17 (30.4) | 0 (0.0) | 0 (0.0) | 2 (11.8) | 10 (58.8) | 4 (23.5) | 0 (0.0) |
| Free care for diabetes (0-18 years) | 44 (41.9) | 12 (27.3) | 0 (0.0) | 0 (0..0) | 1 (8.3) | 6 (50.0) | 2 (16.7) | 0 (0.0) |
| **Category Mean** | **73.4 (69.9)** | **26.0 (34.7)** | **1.8 (6.0)** | **3.3 (11.6)** | **4.6 (16.7)** | **19.5 (70.5)** | **8.4 (30.3)** | **3.5 (12.2)** |
| **Free care on services (1 program)** | | | | | | | | |
| Free care for family planning | 75 (71.4) | 29 (38.7) | 1 (3.4) | 1 (3.4) | 1 (3.4) | 23 (79.3) | 8 (27.6) | 1 (3.4) |
| **Category Mean** | **75.0 (71.4)** | **29.0 (38.7)** | **1.0 (3.4)** | **1.0 (3.4)** | **1.0 (3.4)** | **23.0 (79.3)** | **8.0 (27.6)** | **1.0 (3.4)** |
| **Free care for indigents (2 programs)** | | | | | | | | |
| Free care for abandoned children | 50 (47.6) | 16 (32.0) | 2 (12.5) | 1 (6.3) | 2 (12.5) | 9 (56.3) | 3 (18.8) | 1 (6.3) |
| Free care for indigents | 66 (62.9) | 19 (28.8) | 2 (10.5) | 1 (5.3) | 2 (10.5) | 13 (68.4) | 3 (15.8) | 1 (5.3) |
| **Category Mean** | **58.0 (55.2)** | **17.5 (30.4)** | **2.0 (11.5)** | **1.0 (5.8)** | **2.0 (11.5)** | **11.0 (62.3)** | **3.0 (17.3)** | **1.0 (5.8)** |
| **Budget financing (2 programs)** | | | | | | | | |
| Subvention for care in confessional facilities | 41 (39.0) | 11 (26.8) | 2 (18.2) | 3 (27.3) | 2 (18.2) | 4 (36.4) | 3 (27.3) | 2 (18.2) |
| Budget support for public health facilities | 68 (64.8) | 8 (11.8) | 1 (12.5) | 2 (25.0) | 3 (37.5) | 5 (62.5) | 2 (25.0) | 2 (25.0) |
| **Category Mean** | **54.5 (51.9)** | **9.5 (19.3)** | **1.5 (15.3)** | **2.5 (26.1)** | **2.5 (27.8)** | **4.5 (49.4)** | **2.5 (26.1)** | **2.0 (21.6)** |
| **Budget support targeting a segment of the population (2 programs)** | | | | | | | | |
| Medical evacuation funds (abroad) | 63 (60.0) | 11 (17.5) | 3 (27.3) | 2 (18.2) | 2 (18.2) | 3 (27.3) | 2 (18.2) | 2 (18.2) |
| Subsidized care for civil servants and health personnel | 54 (51.4) | 9 (16.7) | 1 (11.1) | 1 (11.1) | 1 (11.1) | 2 (22.2) | 1 (11.1) | 1 (11.1) |
| **Category Mean** | **58.5 (55.7)** | **10.0 (17.1)** | **2.0 (19.2)** | **1.5 (14.6)** | **1.5 (14.6)** | **2.5 (24.7)** | **1.5 (14.6)** | **1.5 (14.6)** |
| **Prepayment mechanism (4 programs)** | | | | | | | | |
| National health insurance | 59 (56.2) | 10 (16.9) | 4 (40.0) | 1 (10.0) | 1 (10.0) | 2 (20.0) | 2 (20.0) | 1 (10.0) |
| Social security | 70 (66.7) | 12 (17.1) | 2 (16.7) | 1 (8.3) | 1 (8.3) | 3 (25.0) | 1 (8.3) | 1 (8.3) |
| Private health insurance | 81 (77.1) | 22 (27.2) | 2 (9.1) | 3 (13.6) | 1 (4.5) | 13 (59.1) | 3 (13.6) | 3 (13.6) |
| Mutual health organization | 82 (78.1) | 14 (17.1) | 0 (0.0) | 1 (7.1) | 0 (0.0) | 2 (14.3) | 1 (7.1) | 1 (7.1) |
| **Category Mean** | **73.0 (69.5)** | **14.5 (19.6)** | **2.0 (16.4)** | **1.5 (9.8)** | **0.8 (5.7)** | **5.0 (29.6)** | **1.8 (12.3)** | **1.5 (9.8)** |
| **Results based financing (2 programs)** | | | | | | | | |
| Voucher | 61 (58.1) | 13 (21.3) | 1 (7.7) | 2 (15.4) | 1 (7.7) | 2 (15.4) | 3 (23.1) | 1 (7.7) |
| Performance based financing | 91 (86.7) | 37 (40.7) | 5 (13.5) | 6 (16.2) | 8 (21.6) | 28 (75.7) | 14 (37.8) | 8 (21.6) |
| **Category Mean** | **76.0 (72.4)** | **25.0 (31.0)** | **3.0 (10.6)** | **4.0 (15.8)** | **4.5 (14.7)** | **15.0 (45.5)** | **8.5 (30.5)** | **4.5 (14.7)** |
| **Payment at the point of service (1 program)** | | | | | | | | |
| Out of pocket payment | 86 (81.9) | 19 (22.1) | 2 (10.5) | 3 (15.8) | 2 (10.5) | 7 (36.8) | 3 (15.8) | 3 (15.8) |
| **Category Mean** | **86.0 (81.9)** | **19.0 (22.1)** | **2.0 (10.5)** | **3.0 (15.8)** | **2.0 (10.5)** | **7.0 (36.8)** | **3.0 (15.8)** | **3.0 (15.8)** |
|  | | | | | | | | |
| **Overall Mean** | **68.7 (65.4)** | **19.0 (28.1)** | **1.8 (10.8)** | **2.2 (12.1)** | **2.4 (12.8)** | **11.2 (50.3)** | **4.7 (22.0)** | **2.3 (11.9)** |
