# Supplementary material for: Knowledge, attitude, and practices of stakeholders involved in healthcare financing programs on economic evaluations in Cameroon
Source: PLOS Glob Public Health. 2024 Apr 25;4(4):e0003101. doi: 10.1371/journal.pgph.0003101 (PMC11045103; doi:10.1371/journal.pgph.0003101)
Supplement: S2 Table — (DOCX) [file pgph.0003101.s004.docx]

Participants’ level of involvement in a health economic evaluation of Cameroon’s healthcare financing programs (N=106)

| **Healthcare financing programs** | **Some level of involvement** –  n (% selected) | **Level of involvement in a health economic evaluation** – n (% selected out of those who indicated some level of involvement) | | | | | |
| --- | --- | --- | --- | --- | --- | --- | --- |
|  |  | Initial and theoretical design | Design in terms of consultation/discussion with donors | Implementation in terms of coordination | Implementation on the ground | Data analysis | Improvement |
| **Free/subsidy policy focusing on disease control for the entire population (8 programs)** | | | | | | | |
| Subsidized treatment for diabetes | 21 (19.8) | 1 (4.8) | 1 (4.8) | 0 (0.0) | 1 (4.8) | 1 (4.8) | 0 (0.0) |
| Free care for epilepsy | 20 (18.9) | 0 (0.0) | 0 (0.0) | 0 (0.0) | 0 (0.0) | 0 (0.0) | 0 (0.0) |
| Free care for preventive treatment of onchocerciasis | 21 (19.8) | 0 (0.0) | 0 (0.0) | 0 (0.0) | 0 (0.0) | 0 (0.0) | 0 (0.0) |
| Free care for HIV/AIDS | 13 (12.3) | 1 (7.7) | 1 (7.7) | 0 (0.0) | 1 (7.7) | 1 (7.7) | 0 (0.0) |
| Free treatment for tuberculosis | 15 (14.2) | 0 (0.0) | 0 (0.0) | 0 (0.0) | 0 (0.0) | 0 (0.0) | 0 (0.0) |
| Free treatment for leprosy | 21 (19.8) | 0 (0.0) | 0 (0.0) | 0 (0.0) | 0 (0.0) | 0 (0.0) | 0 (0.0) |
| Free treatment for Buruli ulcer | 21 (19.8) | 0 (0.0) | 0 (0.0) | 0 (0.0) | 0 (0.0) | 0 (0.0) | 0 (0.0) |
| Subsidized treatment for cancer | 20 (18.9) | 0 (0.0) | 0 (0.0) | 0 (0.0) | 0 (0.0) | 0 (0.0) | 0 (0.0) |
| **Category Mean** | **19.0 (17.9)** | **0.3 (1.6)** | **0.3 (1.6)** | **0.0 (0.0)** | **0.3 (1.6)** | **0.3 (1.6)** | **0.0 (0.0)** |
| **Free/subsidy policy focusing on controlling a disease targeting part of the population (8 programs)** | | | | | | | |
| Free malaria treatment for children under 5 years old | 13 (12.3) | 1 (7.7) | 1 (7.7) | 1 (7.7) | 1 (7.7) | 1 (7.7) | 1 (7.7) |
| Subsidized malaria treatment for children over 5 years old and adults | 15 (14.2) | 1 (6.7) | 1 (6.7) | 1 (6.7) | 1 (6.7) | 1 (6.7) | 1 (6.7) |
| Free intermittent preventing treatment (IPT) for pregnant women | 15 (14.2) | 0 (0.0) | 0 (0.0) | 0 (0.0) | 0 (0.0) | 0 (0.0) | 0 (0.0) |
| Free long lasting insecticidal (LLI) bed nets | 21 (19.8) | 0 (0.0) | 0 (0.0) | 0 (0.0) | 0 (0.0) | 0 (0.0) | 0 (0.0) |
| Free care for malnutrition | 20 (18.9) | 0 (0.0) | 0 (0.0) | 0 (0.0) | 0 (0.0) | 0 (0.0) | 0 (0.0) |
| Free treatment for intestinal helminthiasis | 0 (0.0) | - | - | - | - | - | - |
| Free treatment for schistosomiasis | 20 (18.9) | 0 (0.0) | 0 (0.0) | 0 (0.0) | 0 (0.0) | 0 (0.0) | 0 (0.0) |
| Free care for diabetes (0-18 years) | 20 (18.9) | 0 (0.0) | 0 (0.0) | 0 (0.0) | 0 (0.0) | 0 (0.0) | 0 (0.0) |
| **Category Mean** | **15.5 (14.7)** | **0.3 (2.1)** | **0.3 (2.1)** | **0.3 (2.1)** | **0.3 (2.1)** | **0.3 (2.1)** | **0.3 (2.1)** |
| **Free care on services (1 program)** | | | | | | | |
| Free care for family planning | 0 (0.0) | - | - | - | - | - | - |
| **Category Mean** | **0.0 (0.0)** | **-** | **-** | **-** | **-** | **-** | **-** |
| **Free care for indigents (2 programs)** | | | | | | | |
| Free care for abandoned children | 18 (17.0) | 1 (5.6) | 1 (5.6) | 0 (0.0) | 0 (0.0) | 0 (0.0) | 0 (0.0) |
| Free care for indigents | 19 (17.9) | 1 (5.3) | 1 (5.3) | 0 (0.0) | 0 (0.0) | 0 (0.0) | 0 (0.0) |
| **Category Mean** | **18.5 (17.5)** | **1.0 (5.5)** | **1.0 (5.5)** | **0.0 (0.0)** | **0.0 (0.0)** | **0.0 (0.0)** | **0.0 (0.0)** |
| **Budget financing (2 programs)** | | | | | | | |
| Subvention for care in confessional facilities | 20 (18.9) | 0 (0.0) | 0 (0.0) | 0 (0.0) | 0 (0.0) | 0 (0.0) | 0 (0.0) |
| Budget support for public health facilities | 20 (18.9) | 0 (0.0) | 0 (0.0) | 0 (0.0) | 0 (0.0) | 0 (0.0) | 0 (0.0) |
| **Category Mean** | **20.0 (18.9)** | **0.0 (0.0)** | **0.0 (0.0)** | **0.0 (0.0)** | **0.0 (0.0)** | **0.0 (0.0)** | **0.0 (0.0)** |
| **Budget support targeting a segment of the population (2 programs)** | | | | | | | |
| Medical evacuation funds (abroad) | 20 (18.9) | 0 (0.0) | 0 (0.0) | 0 (0.0) | 0 (0.0) | 0 (0.0) | 0 (0.0) |
| Subsidized care for civil servants and health personnel | 19 (17.9) | 0 (0.0) | 0 (0.0) | 0 (0.0) | 0 (0.0) | 0 (0.0) | 0 (0.0) |
| **Category Mean** | **19.5 (18.4)** | **0.0 (0.0)** | **0.0 (0.0)** | **0.0 (0.0)** | **0.0 (0.0)** | **0.0 (0.0)** | **0.0 (0.0)** |
| **Prepayment mechanism (4 programs)** | | | | | | | |
| National health insurance | 21 (19.8) | 0 (0.0) | 0 (0.0) | 0 (0.0) | 0 (0.0) | 0 (0.0) | 0 (0.0) |
| Social security | 21 (19.8) | 0 (0.0) | 0 (0.0) | 0 (0.0) | 0 (0.0) | 0 (0.0) | 0 (0.0) |
| Private health insurance | 18 (17.0) | 0 (0.0) | 0 (0.0) | 0 (0.0) | 0 (0.0) | 0 (0.0) | 0 (0.0) |
| Mutual health organization | 21 (19.8) | 0 (0.0) | 0 (0.0) | 0 (0.0) | 0 (0.0) | 0 (0.0) | 0 (0.0) |
| **Category Mean** | **20.3 (19.1)** | **0.0 (0.0)** | **0.0 (0.0)** | **0.0 (0.0)** | **0.0 (0.0)** | **0.0 (0.0)** | **0.0 (0.0)** |
| **Results based financing (2 programs)** | | | | | | | |
| Voucher | 19 (17.9) | 1 (5.3) | 0 (0.0) | 0 (0.0) | 0 (0.0) | 0 (0.0) | 0 (0.0) |
| Performance based financing | 15 (14.2) | 0 (0.0) | 0 (0.0) | 0 (0.0) | 0 (0.0) | 0 (0.0) | 0 (0.0) |
| **Category Mean** | **17.0 (16.1)** | **0.5 (2.7)** | **0.0 (0.0)** | **0.0 (0.0)** | **0.0 (0.0)** | **0.0 (0.0)** | **0.0 (0.0)** |
| **Payment at the point of service (1 program)** | | | | | | | |
| Out of pocket payment | 21 (19.8) | 0 (0.0) | 0 (0.0) | 0 (0.0) | 0 (0.0) | 0 (0.0) | 0 (0.0) |
| **Category Mean** | **21.0 (19.8)** | **0.0 (0.0)** | **0.0 (0.0)** | **0.0 (0.0)** | **0.0 (0.0)** | **0.0 (0.0)** | **0.0 (0.0)** |
|  | | | | | | | |
| **Overall Mean** | **16.8 (15.8)** | **0.3 (1.5)** | **0.2 (1.1)** | **0.04 (0.3)** | **0.1 (0.5)** | **0.1 (0.5)** | **0.04 (0.3)** |
