# Supplementary material for: Knowledge, attitude, and practices of stakeholders involved in healthcare financing programs on economic evaluations in Cameroon
Source: PLOS Glob Public Health. 2024 Apr 25;4(4):e0003101. doi: 10.1371/journal.pgph.0003101 (PMC11045103; doi:10.1371/journal.pgph.0003101)
Supplement: S3 Table — (DOCX) [file pgph.0003101.s005.docx]

Participants’ knowledge of whether Cameroon’s healthcare financing programs have been the subject of economic evaluation (N=106)

| **Healthcare financing programs** | **Don’t know if the program has ever been the subject of economic evaluation, or if it is planned** – n (% selected) | **Has never been the subject of economic evaluation, and it is not planned** – n (% selected out of those who know if the program has ever been the subject of economic evaluation, of it is planned) | **Has already been the subject of …**  n (% selected out of those who indicated that the program has been the subject of economic evaluation) | | | | | | |
| --- | --- | --- | --- | --- | --- | --- | --- | --- | --- |
|  |  |  | Cost analysis | Effects/  consequences analysis | Cost- minimization analysis | Cost-effectiveness analysis | Cost-utility analysis | Cost-benefit analysis | Cost-consequences analysis |
| **Free/subsidy policy focusing on disease control for the entire population (8 programs)** | | | | | | | | | |
| Subsidized treatment for diabetes | 8 (7.5) | 96 (98.0) | 2 (100) | 0 (0.0) | 0 (0.0) | 0 (0.0) | 0 (0.0) | 0 (0.0) | 0 (0.0) |
| Free care for epilepsy | 6 (5.7) | 95 (95.0) | 3 (60.0) | 0 (0.0) | 0 (0.0) | 0 (0.0) | 0 (0.0) | 0 (0.0) | 0 (0.0) |
| Free care for preventive treatment of onchocerciasis | 8 (7.5) | 96 (98.0) | 2 (100.0) | 1 (50.0) | 0 (0.0) | 0 (0.0) | 0 (0.0) | 0 (0.0) | 0 (0.0) |
| Free care for HIV/AIDS | 13 (12.3) | 88 (94.6) | 4 (80.0) | 1 (20.0) | 0 (0.0) | 1 (20.0) | 0 (0.0) | 0 (0.0) | 0 (0.0) |
| Free treatment for tuberculosis | 13 (12.3) | 89 (95.7) | 3 (75.0) | 0 (0.0) | 0 (0.0) | 0 (0.0) | 0 (0.0) | 0 (0.0) | 0 (0.0) |
| Free treatment for leprosy | 5 (4.7) | 96 (95.0) | 3 (60.0) | 0 (0.0) | 0 (0.0) | 0 (0.0) | 0 (0.0) | 0 (0.0) | 0 (0.0) |
| Free treatment for Buruli ulcer | 7 (6.6) | 96 (97.0) | 2 (66.7) | 0 (0.0) | 0 (0.0) | 0 (0.0) | 0 (0.0) | 0 (0.0) | 0 (0.0) |
| Subsidized treatment for cancer | 6 (5.7) | 97 (97.0) | 2 (66.7) | 0 (0.0) | 0 (0.0) | 0 (0.0) | 0 (0.0) | 0 (0.0) | 0 (0.0) |
| **Category Mean** | **8.3 (7.8)** | **94.1 (96.3)** | **2.6 (76.1)** | **0.3 (8.8)** | **0.0 (0.0)** | **0.1 (2.5)** | **0.0 (0.0)** | **0.0 (0.0)** | **0.0 (0.0)** |
| **Free/subsidy policy focusing on controlling a disease targeting part of the population (8 programs)** | | | | | | | | | |
| Free malaria treatment for children under 5 years old | 15 (14.2) | 88 (96.7) | 3 (100.0) | 1 (33.3) | 0 (0.0) | 1 (33.3) | 0 (0.0) | 0 (0.0) | 0 (0.0) |
| Subsidized malaria treatment for children over 5 years old and adults | 15 (14.2) | 89 (97.8) | 2 (100.0) | 1 (50.0) | 0 (0.0) | 1 (50.0) | 0 (0.0) | 0 (0.0) | 0 (0.0) |
| Free intermittent preventing treatment (IPT) for pregnant women | 13 (12.3) | 91 (97.8) | 2 (100.0) | 1 (50.0) | 0 (0.0) | 1 (50.0) | 0 (0.0) | 0 (0.0) | 0 (0.0) |
| Free long lasting insecticidal (LLI) bed nets | 12 (11.3) | 93 (98.9) | 1 (100.0) | 0 (0.0) | 0 (0.0) | 0 (0.0) | 0 (0.0) | 0 (0.0) | 0 (0.0) |
| Free care for malnutrition | 10 (9.4) | 93 (96.9) | 3 (100.0) | 1 (33.3) | 0 (0.0) | 0 (0.0) | 0 (0.0) | 0 (0.0) | 0 (0.0) |
| Free treatment for intestinal helminthiasis | 9 (8.5) | 94 (96.9) | 3 (100.0) | 1 (33.3) | 0 (0.0) | 1 (33.3) | 0 (0.0) | 0 (0.0) | 0 (0.0) |
| Free treatment for schistosomiasis | 8 (7.5) | 96 (98.0) | 2 (100.0) | 0 (0.0) | 0 (0.0) | 0 (0.0) | 0 (0.0) | 0 (0.0) | 0 (0.0) |
| Free care for diabetes (0-18 years) | 9 (8.5) | 94 (96.9) | 2 (66.7) | 0 (0.0) | 0 (0.0) | 0 (0.0) | 0 (0.0) | 0 (0.0) | 0 (0.0) |
| **Category Mean** | **11.4 (10.7)** | **92.3 (97.5)** | **2.3 (95.8)** | **0.6 (25.0)** | **0.0 (0.0)** | **0.5 (20.8)** | **0.0 (0.0)** | **0.0 (0.0)** | **0.0 (0.0)** |
| **Free care on services (1 program)** | | | | | | | | | |
| Free care for family planning | 9 (8.5) | 94 (96.9) | 2 (66.7) | 2 (66.7) | 0 (0.0) | 0 (0.0) | 0 (0.0) | 0 (0.0) | 0 (0.0) |
| **Category Mean** | **9.0 (8.5)** | **94.0 (96.9)** | **2.0 (66.7)** | **2.0 (66.7)** | **0.0 (0.0)** | **0.0 (0.0)** | **0.0 (0.0)** | **0.0 (0.0)** | **0.0 (0.0)** |
| **Free care for indigents (2 programs)** | | | | | | | | | |
| Free care for abandoned children | 7 (6.6) | 97 (98.0) | 2 (100.0) | 0 (0.0) | 0 (0.0) | 0 (0.0) | 0 (0.0) | 0 (0.0) | 0 (0.0) |
| Free care for indigents | 7 (6.6) | 97 (98.0) | 2 (100.0) | 0 (0.0) | 0 (0.0) | 0 (0.0) | 0 (0.0) | 0 (0.0) | 0 (0.0) |
| **Category Mean** | **7.0 (6.6)** | **97.0 (98.0)** | **2.0 (100.0)** | **0.0 (0.0)** | **0.0 (0.0)** | **0.0 (0.0)** | **0.0 (0.0)** | **0.0 (0.0)** | **0.0 (0.0)** |
| **Budget financing (2 programs)** | | | | | | | | | |
| Subvention for care in confessional facilities | 7 (6.6) | 96 (97.0) | 2 (66.7) | 0 (0.0) | 0 (0.0) | 0 (0.0) | 0 (0.0) | 0 (0.0) | 0 (0.0) |
| Budget support for public health facilities | 10 (9.4) | 95 (99.0) | 0 (0.0) | 0 (0.0) | 0 (0.0) | 0 (0.0) | 0 (0.0) | 0 (0.0) | 0 (0.0) |
| **Category Mean** | **8.5 (8.0)** | **95.5 (98.0)** | **1.0 (33.4)** | **0.0 (0.0)** | **0.0 (0.0)** | **0.0 (0.0)** | **0.0 (0.0)** | **0.0 (0.0)** | **0.0 (0.0)** |
| **Budget support targeting a segment of the population (2 programs)** | | | | | | | | | |
| Medical evacuation funds (abroad) | 6 (5.7) | 97 (97.0) | 2 (66.7) | 0 (0.0) | 0 (0.0) | 0 (0.0) | 0 (0.0) | 0 (0.0) | 0 (0.0) |
| Subsidized care for civil servants and health personnel | 8 (7.5) | 96 (98.0) | 1 (50.0) | 0 (0.0) | 0 (0.0) | 0 (0.0) | 0 (0.0) | 0 (0.0) | 0 (0.0) |
| **Category Mean** | **7.0 (6.6)** | **96.5 (97.5)** | **1.5 (58.4)** | **0.0 (0.0)** | **0.0 (0.0)** | **0.0 (0.0)** | **0.0 (0.0)** | **0.0 (0.0)** | **0.0 (0.0)** |
| **Prepayment mechanism (4 programs)** | | | | | | | | | |
| National health insurance | 6 (5.7) | 97 (97.0) | 2 (66.7) | 0 (0.0) | 0 (0.0) | 0 (0.0) | 0 (0.0) | 0 (0.0) | 0 (0.0) |
| Social security | 8 (7.5) | 97 (99.0) | 1 (100.0) | 0 (0.0) | 0 (0.0) | 0 (0.0) | 0 (0.0) | 0 (0.0) | 0 (0.0) |
| Private health insurance | 11 (10.4) | 94 (98.9) | 1 (100.0) | 0 (0.0) | 0 (0.0) | 0 (0.0) | 0 (0.0) | 0 (0.0) | 0 (0.0) |
| Mutual health organization | 7 (6.6) | 98 (99.0) | 1 (100.0) | 0 (0.0) | 0 (0.0) | 0 (0.0) | 0 (0.0) | 0 (0.0) | 0 (0.0) |
| **Category Mean** | **8.0 (7.6)** | **96.5 (98.5)** | **1.3 (91.7)** | **0.0 (0.0)** | **0.0 (0.0)** | **0.0 (0.0)** | **0.0 (0.0)** | **0.0 (0.0)** | **0.0 (0.0)** |
| **Results based financing (2 programs)** | | | | | | | | | |
| Voucher | 9 (8.5) | 96 (99.0) | 1 (100.0) | 0 (0.0) | 0 (0.0) | 0 (0.0) | 0 (0.0) | 0 (0.0) | 0 (0.0) |
| Performance based financing | 18 (17.0) | 87 (98.9) | 1 (100.0) | 0 (0.0) | 0 (0.0) | 0 (0.0) | 0 (0.0) | 0 (0.0) | 0 (0.0) |
| **Category Mean** | **13.5 (12.8)** | **91.5 (99.0)** | **1.0 (100.0)** | **0.0 (0.0)** | **0.0 (0.0)** | **0.0 (0.0)** | **0.0 (0.0)** | **0.0 (0.0)** | **0.0 (0.0)** |
| **Payment at the point of service (1 program)** | | | | | | | | | |
| Out of pocket payment | 9 (8.5) | 94 (96.9) | 1 (33.3) | 0 (0.0) | 0 (0.0) | 0 (0.0) | 0 (0.0) | 0 (0.0) | 0 (0.0) |
| **Category Mean** | **9.0 (8.5)** | **94.0 (96.9)** | **1.0 (33.3)** | **0.0 (0.0)** | **0.0 (0.0)** | **0.0 (0.0)** | **0.0 (0.0)** | **0.0 (0.0)** | **0.0 (0.0)** |
|  | | | | | | | | | |
| **Overall Mean** | **9.1 (8.6)** | **94.6 (97.6)** | **1.6 (72.8)** | **0.3 (11.2)** | **0.0 (0.0)** | **0.1 (2.6)** | **0.0 (0.0)** | **0.0 (0.0)** | **0.0 (0.0)** |
